# Unfolding and dynamics of affect bursts decoding in humans

**DOI:** 10.1371/journal.pone.0206216

**Published:** 2018-10-30

**Authors:** Simon Schaerlaeken, Didier Grandjean

**Affiliations:** Neuroscience of Emotion and Affective Dynamics Lab, Faculty of Psychology and Educational Sciences and Swiss Center for Affective Sciences, University of Geneva, Geneva, Switzerland; Harvard Medical School, UNITED STATES

## Abstract

The unfolding dynamics of the vocal expression of emotions are crucial for the decoding of the emotional state of an individual. In this study, we analyzed how much information is needed to decode a vocally expressed emotion using affect bursts, a gating paradigm, and linear mixed models. We showed that some emotions (fear, anger, disgust) were significantly better recognized at full-duration than others (joy, sadness, neutral). As predicted, recognition improved when greater proportion of the stimuli was presented. Emotion recognition curves for anger and disgust were best described by higher order polynomials (second to third), while fear, sadness, neutral, and joy were best described by linear relationships. Acoustic features were extracted for each stimulus and subjected to a principal component analysis for each emotion. The principal components were successfully used to partially predict the accuracy of recognition (i.e., for anger, a component encompassing acoustic features such as fundamental frequency (f0) and jitter; for joy, pitch and loudness range). Furthermore, the impact of the principal components on the recognition of anger, disgust, and sadness changed with longer portions being presented. These results support the importance of studying the unfolding conscious recognition of emotional vocalizations to reveal the differential contributions of specific acoustical feature sets. It is likely that these effects are due to the relevance of threatening information to the human mind and are related to urgent motor responses when people are exposed to potential threats as compared with emotions where no such urgent response is required (e.g., joy).

## Introduction

Communication of emotion is essential in human life. For example, it provides support for social coordination and conflict resolution. Vocal expression in particular is crucial to survival and social relationships [1]. Researchers have been increasingly interested in studying emotions by using vocalization and sounds since the technological means of storing and reproducing voice sounds became available to psychologists [2]. Seventy years of research in vocal expressions have demonstrated that listeners (i.e., decoders) reliably and accurately perceive emotions in the voices of human subjects (i.e., encoders) at rates that are six-fold better than chance (e.g., for review, see [3]). It is also apparent that some emotions are better recognized than others from vocal cues [2, 4, 5]. In these previous experiments, vocalizations of anger, for example, were very well recognized while the ones trying to convey disgust were poorly detected. This pioneer work was usually based on relatively long speech samples (i.e., sentences of approximately 1–2 s in duration) raising two crucial research questions: 1) In addition to being more accurately detected, some emotions might be recognized faster than others within those long stimuli. Consequently, one could be interested in how emotion recognition unfolds over time. 2) Long speech samples might be less appropriate for studying emotions such as disgust or surprise which people typically express via a different type of vocalization such as affect bursts. Therefore, one could also be interested in how well emotions are recognized using such type of vocal cues.

Speaking of the temporal aspect of emotion recognition is indeed essential since the vocalization of emotions and its prosodic structure are intrinsically dynamic (unlike facial expressions, which can be tested in static form, although their occurrences in everyday life are definitively dynamic as well). Therefore, to characterize when vocal emotions are processed by the cognitive system and appear to be recognized, one must consider the timing information. Given the variability of the underlying temporal properties of vocal expressions, it is possible that discrete emotions unfold at different rates and are thus recognized from the prosody at different points in time [6]. On one hand, the accurate recognition of vocal emotions allows individual to react adequately to specific emotionally connoted behaviors or at least activating specific action tendencies [7]. This is emphasized by the biological and social significance of coordinating emotional behaviors in human communication. On the other hand, recognizing some emotions faster than others represents an evolutionary advantage. Emotions seem to motivate people to respond quickly and adequately to stimuli in the environment, improving survival chances and allowing them to avoid danger or to detect relevant important information in surroundings, according to the evolutionary theory of emotions [8]. For example, in the case of vocal expression of anger or fear, reacting timely and appropriately to such vocal cues in life-threatening situations can make a big difference. Neural mechanisms, cognitive responses, and action tendencies when presented with aversive and life-threatening stimuli have already been studied for both anger and fear using facial expression and vocal cues [9–11]. The temporal aspect of such vocal cues remains to be further explored. Controlling the duration of the stimuli that the listener is exposed to gives us a way to control the amount of information available by the cognitive system of the listener. By varying the portion of the stimuli presented and assessing emotion recognition at every step, one could investigate both qualitative and quantitative aspects. On the one hand, qualitative information refers to how accurate and confident listeners are about the presence of discrete emotions. On the other hand, quantitative information describes how much acoustic variation is needed for specific emotions to be accurately recognized. One way to combine both qualitative and quantitative aspect to study the impact of the temporal aspect of emotion recognition is to use a gating paradigm. This type of paradigm has been successfully used to estimate the temporal course of operations leading to recognition of auditory events [12]. In gating studies, auditory “gates” are constructed as a function of specific time increments, or linguistic units of spoken language. They are then presented to listeners in segments of increasing duration starting at the beginning of the relevant stimulus. The last gate usually corresponds to the entire stimulus event (see [13], for an overview of design issues). Ultimately, researchers can estimate the “identification point” of specific target meanings by locating the gate at which the target is accurately recognized by a participant without further changes at longer gate durations for the same stimulus [14, 15].

Pell and Kotz (2011) used such a gating paradigm to study the recognition of different emotions over the time course of an utterance [16]. They estimated how much vocal information was needed for listeners to categorize five basic emotions. Using semantically anomalous pseudo-utterances, they showed that anger, sadness, fear, and neutral expressions were recognized more accurately at short gate intervals than joy and particularly disgust. However, as speech unfolded, recognition of joy improved significantly towards the end of the utterance. At each gate fear was recognized more accurately than other emotions. The results also indicated different identification points for different emotions: fear (*M* = 517*ms*), sadness (*M* = 576*ms*), and neutral (*M* = 510*ms*) expressions were identified from shorter acoustic events than the other emotions. Pell and Kotz showed that participants needed more information to decode vocal expressions of joy and disgust, whereas anger, fear, and neutral expressions were recognized more accurately at the lower gates [16]. Cornew, Carver, and Love (2009) reported similar results while computing identification points [17]. They showed that identification points differed for each emotion as the gated pseudo-utterance unfolded. Participants recognized neutral sentences quickly and accurately (*M* = 444*ms*) followed by angry (*M* = 723*ms*), and finally happy (*M* = 802*ms*) sentences. However, although identification points were computed, less is known about the shape of the recognition curves. These curves would reflect the evolution of discrete emotion recognition as a function of gate duration. As more information accumulates through the unfolding of the emotional utterance, the recognition accuracy increases and emotions are more accurately decoded. It is conceivable that the emotion recognition is characterized by a nonlinear relationship with the gate duration. The differences in the time course of recognizing vocal expressions suggest different function shapes for the recognition curves. In addition to defining identification points, studying the relationship between timing and accuracy of recognition with the shape of recognition curves would allow us to obtain a more precise continuous comparison between emotions. Furthermore, such analysis could give us insights into the emotion-specific critical period of time when recognition is most improved by addition of auditory information, leading to a better appreciation of emotion recognition as a whole.

Emotions expressed through vocalization can take many forms. When studying emotional prosody, one must acknowledge the rich and wide range of emotional signals present in the human voice. Many studies have focused on specific aspects of it by, for example, studying teasing [18], varieties of laughter [19], and motherese [20]. Some of these signals are unique to specific emotions such as laughter and happiness. Therefore, in an attempt to be inclusive, many researchers aiming to compare the recognition of different emotions focused on portrayed emotions using words, pseudo-utterances, or meaningless sentences (see review [21]). However, some emotions, such as disgust or surprise, are better expressed through different vocal cues. One of these types of vocalization, affect bursts, achieves high recognition accuracy for all emotions. Affect bursts are defined as “very brief, discrete, nonverbal expressions of affect in both face and voice as triggered by clearly identifiable events” [22]. They are shared with our closest living relatives in the animal kingdom such as chimpanzees and bonobos [23]. Vocal bursts include sounds like shrieks, groans, or grunts. They can express many different states in a fraction of a second, such as awe with the iconic ‘whaaaaaaaa’, the relieved ‘ouuuf’, the disgusted ‘aargh’, the surprised ‘oh!’, or the enthusiastic and even more emblematic ‘yeeee-haaaw’ [22]. Multiple studies have already explored the recognition of different emotions in affect burst demonstrating very hight accuracy. One study focused on recognizing displays of 10 different emotions: admiration, anger, boredom, contempt, disgust, elation, relief, threat, startle, and worry. They reported 81.1% accuracy [24]. A second study reported 70.1% accuracy for vocal bursts for five different positive emotions: achievement, amusement, contentment, pleasure, and relief, across two cultural groups [25]. A last study focusing on 22 different emotions portrayed by affect bursts reported that the correct identification of vocal bursts for anger, disgust, fear, sadness, and surprise ranged from 80% to 96% [26]. The use of different types of vocalization for studying the unfolding of emotion recognition would complete and extend the work started by Pell and Kotz [16].

Unfortunately, while affect bursts are associated with very high accuracy, little is know about the acoustic cues associated with such a wide range of stimuli. Decoding the emotion expressed in the voice of another human being is believed to be based on acoustic attributes modified as the encoder speaks. Patterns of acoustic cues have been predicted to be associated with different emotions [2]. The perceptive system uses the amount of variation over time of a speaker’s acoustical characteristics to build up a probabilistic categorical representation based on the perceptual dynamics. For example, loudness (which is based on the energy), vocal pitch (based on the fundamental frequency), and other acoustic features are used to evaluate the emotion expressed as speech unfolds [27]. Acoustic features—such as F0 mean or range, energy mean or range, and speech rate—vary continuously with emotional arousal (see [21] for a review of this work). For instance, the pitch, based on the fundamental frequency, can be used to differentiate emotions with low arousal (such as sadness, joy, and anxiety) from emotions with high arousal (despair, elation, panic fear, and hot anger). Emotions with low arousal have a lower fundamental frequency and pitch. Such acoustic features are included in the Geneva Minimal Acoustic Parameter Set (GEMAPS) developed specifically for voice research and affective computing [28]. To our current knowledge, this set of acoustic characteristics have not been computed on affect bursts and much is left to be explored about the acoustic features driving the accurate categorization of such vocal cues.

All in all, while affect bursts represent a strong alternative to longer stimuli when it comes to emotion recognition, a lot is left unexplored. Little is known about the amount of information necessary to correctly classify affect bursts. Recognition curves of such emotional signal have never been explored and acoustic features associated with them could be further investigated. We aimed to utilize advantages of both powerful statistical methods with unbiased ratings and a gating paradigm to explore the different time courses of emotion recognition. We predicted that negative emotions and those inducing an urgent reaction would be recognized earlier than neutral or non-urgent emotions in affect bursts. Moreover, emotions usually displayed using affect bursts in everyday life such as disgust would show a faster and more accurate recognition. Our goal was also to highlight the acoustic features associated with the shared representation of specific emotions. We further hypothesize that emotion recognition is dependent on acoustic features and that some acoustic features might have a differential impact at different gates. Overall, we aimed to underline the importance of studying the time course of emotion recognition for affect bursts.

## Materials and methods

### Participants

Eighty participants took part in this study (62 females, 18 males). The majority of them spoke French as their first language, with only four of them speaking English. The average age was 23.08 years (*SD* = 7.19) and most participants were psychology students. The study was approved by the ethics commission of the department of Psychology and Educational Sciences at the University of Geneva.

### Stimuli

The stimuli used in this study came from digital recordings of different affect bursts using only the sound of the letter A (*/aaa/*). The recordings were produced by actors and actresses, both genders being represented equally (five females, five males). They are part of the GEneva Multimodal Emotion Portrayals (GEMEP) database [29]. These constrained affect bursts were used in this experiment to avoid any linguistic and/or semantic bias. Five emotions were selected for this study: anger, disgust, fear, joy, and sadness. These emotions are thought to show discrete forms of expression in the face and voice. Surprise was excluded from this list because of the difficulty in simulating a realistic expression experimentally. Neutral recordings were also used. We selected 20 recordings from the database for each emotion (except for disgust, which is represented through only 10 recordings because of the poor quality of other recordings). Each recording represented uniquely one of the emotions selected for this study. The emotion expressed in each recording did not change over the course of the recording and was unambiguous. The affect bursts were processed with Praat, version 5.3.51 [30]. The volume of each recording was normalized to maintain a uniform listening experience between different stimuli (with a coefficient counterbalancing the average and maximum energy) and silences at the beginning and at the end of each recording were removed. Each complete recording were cut into smaller chunks with an incremental duration of 50 ms. It is important to acknowledge that each emotions was represented by different recordings varying greatly in length. In our dataset, the smallest recording generated four subdivisions while the longest generated 101 subdivisions (S1 Table). All the newly created chunks constituted the stimulus set used for this study. The shortest resulting stimuli last 50 ms and the longest 5,050 ms. This process generated 8,400 stimuli. Due to the great number of stimuli, each participant only saw a fraction of the whole. We generated lists of 105 stimuli picked from the whole dataset and presented them in a pseudo-random order. Three or four different participants evaluated each stimulus. Amongst the 8,400 stimuli, we picked 72 to be presented more frequently (10 times) to the participants in order to test the reliability of emotional judgments across the different individuals.

### Experimental procedure

Participants completed the experiment on a computer. The experiment itself was programmed with Windev, version 15 (PC SOFT, http://www.windev.com) and ran on computers with a screen resolution of 1, 280 × 1, 024 pixels. Volume was set to 50% and could be adjusted by the participant. Headphones were provided. The experiment lasted around 30 min. The participants had to sign an informed consent form presented in a written form and completed a demographic questionnaire before starting the experiment. The instructions were displayed on the computer screen and a set of three practice trials was presented to the participant (with stimuli not used in the main task). A unique list of 105 stimuli out of the entire dataset was then presented in a pseudo-random order for each participant. The participant listened to each stimulus and then rated six emotions (anger, fear, joy, sadness, disgust, surprise) and neutral with seven corresponding sliders from 0 to 100. Multiple emotion sliders could be moved but at least one of them had to be moved. The participants were also asked to rate their confidence level for the choice they made. The emotions and confidence levels were evaluated on a continuous scale. This process was repeated for the 105 stimuli used per participant. Finally, the data was anonymised based on the ethics commission’s requirements (raw data is provided in S1 Appendix).

### Statistical analysis

Analyses were performed on data from all 80 participants using the programming language R version 3.2.1, R Studio version 1.0.136, and the package *lme4* version 1.1-17 [31]. First, we grouped stimuli into bins representing the percentage of the total duration presented. In doing so, the different emotions were represented on the same scale independent of their global duration. We created two sets of bins: a general one at 25% intervals (25%-50%-75%-100%) and a more detailed one with 10% intervals (10%-20%-….-90%-100%). A bin grouped together multiple “gates,” as defined when we cut the original recordings. However, for the sake of using the same terminology here as in the studies that use a gating paradigm, we also used the term “gate” to represent the bins in the analysis. Preliminary analyses of our data showed a strong choice bias towards neutral responses at the early gate (further details in S2 Fig). We decided to compute the unbiased hit rate (Hu score) for every emotion at every 10% gate [32]. In order to do so, we first defined a trial as successfully recognized when the highest value of the continuous emotional scales corresponded to the emotion presented. With this information, we computed a confusion matrix created based on the hits and misses of all the participants for each emotion at every 10% gates (S2 Table). This confusion matrix was used to compute the Hu scores. We also computed these unbiased scores, at the participant level, based on a personal confusion matrix computed for every emotion at every 25% gate. Only the cruder gates were used for this case to provide enough data for the computation of Hu scores. Since each participant was associated with his/her own set of Hu scores, this provided us with enough variability at every 25% gate to compute statistical models. We used these unbiased scores for each participant to examine the accuracy of the recognition of emotions at each gate and for each emotion using linear mixed models (LMMs). LMMs are extension of linear regression models taking advantage of the computation of random effects [33]. We used chi-square difference tests to investigate the contribution of each variable and their interaction. We report the effect sizes in accordance with the approach of Nakagawa and Schielzeth (2013), implemented in the “MuMIn” R package [34]. They developed an approach based on two indicators, a marginal and a conditional *R*^2^($R_{m}^{2}$ and $R_{c}^{2}$, respectively), allowing comparability with standard methods, while taking into account the variance explained by the random effects. $R_{m}^{2}$ is the variance explained by the fixed factors, whereas $R_{c}^{2}$ is the variance explained by the entire model (both fixed and random effects). We calculated them for each effect in our statistical models. The fixed effects for our main model were the emotion expressed (anger, sadness, joy, disgust, fear, neutral) and the duration of the gates (25-50-75-100%). The random intercepts effects encapsulated the variability related to each participant. We used a step-up strategy while building the model to test the different combinations of fixed effects. We also computed a polynomial contrast for each recognition curve to determine the nonlinear shape of the curve over time. For the 10 gates model, we computed polynomial regression and the corresponding root mean square error (RMSE). Finally, we estimated a set of 42 acoustical features proposed by the Geneva Minimalist Acoustic Parameter Set, developed for emotional prosody research [28]. Dimensionality reduction was applied through a principal component analysis (PCA) specific to each emotion. We selected four components for each emotion based on the cumulative sum of eigenvalues (S3 Table). These components were later used as fixed effect in LMM to model the impact of acoustic features on emotion recognition.

For completeness, the detailed protocol used in this study can found at http://dx.doi.org/10.17504/protocols.io.rr2d58e.

## Results

Over the 8,400 trials, the emotions expressed were recognized 43.11% of the time (*ICC* = 0.75). On average, the certainty level reached 52.26%. Based on a binomial distribution (recognized/not recognized), we computed a generalized LMM comparing the emotion expressed and the emotion selected by the participants. The general idea was to determine if participants preferentially chose the emotion depicted by the stimulus over the other options independently of duration. For example, the model computed the percentage of the response “anger”, “fear”, “disgust”, “joy”, “neutral”, and “sadness” chosen by the participants for stimuli expressing fear. If participants correctly recognized fearful stimuli, the percentage of responses for “fear” would be greater than for the other emotions. In order to do so, we used the interaction between all the possible emotions depicted in our stimuli and all the possible emotions the participants could choose from as the fixed effects for our model. We compared this model encompassing the interaction between stimulus emotions and emotional scales to a model with only the main effects. This analysis revealed that the interaction significantly improved the explained variance, *χ*^2^(43, *N* = 8, 400) = 10, 385, *p* < 0.001, $R_{m}^{2}=0.369$, $R_{c}^{2}=0.369$ (S1 Fig). When contrasting the choice of one particular emotional scale with the rest of the scales presented, we demonstrated that participants chose the corresponding scale for each emotion presented over the other scales at a significant level (S4 Table). Furthermore, independent of duration, the different emotions were recognized with different accuracy (Table 1). Finally, gender did not seem to affect emotion recognition in this study (S2 Appendix).

**Table 1:** Percentages of recognition and confidence for each emotion across all stimuli.

	Anger	Disgust	Fear	Joy	Neutral	Sadness
Recognition%	72.86	67.51	60.87	21.67	33.37	32.09
Certainty%	61.78	57.59	53.56	51.04	45.95	48.51

### Recognition accuracy

**Fig 1:**
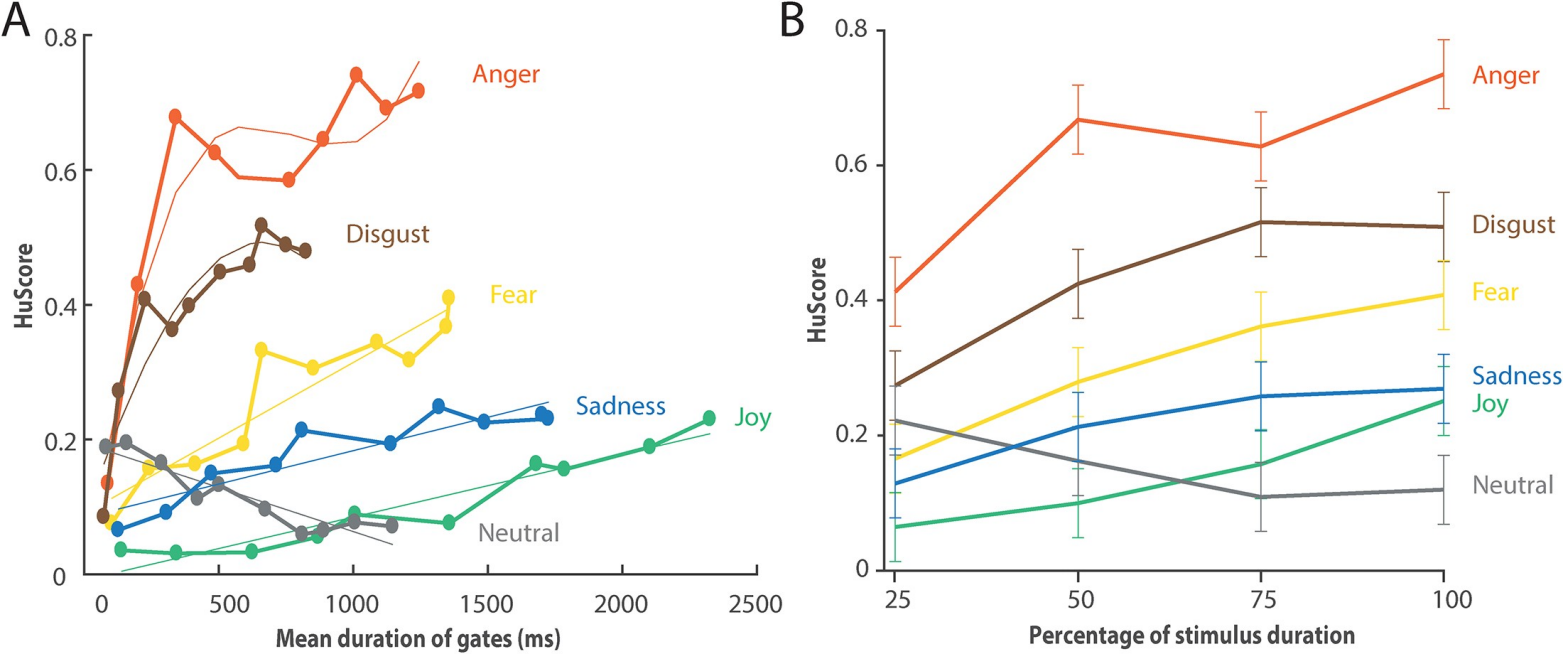
Unbiased recognition over time. (**A**) Graphic of the unbiased ratings for every emotion at every gate. Shown is the unbiased estimated percentage of correct recognition of the emotion expressed for different lengths of stimulus presented (divided into 10 gates representing the percentage of the full stimulus presented, e.g., 10%, 20%…90%, and 100%). For example, 100% for Anger correspond to a group of stimuli with varying length ranging from 400 to 2250 that represent at least 90.1% of the original corresponding stimulus. The mean duration for this group corresponds to 1244 and is used to represent this specific gate. The recognition percentages were computed with the confusion matrix based on continuous responses from participants. Continuous thinner lines represent the best polynomial fit based on the orthogonal polynomial contrasts and RMSE performed in Table 3 (anger: cubic, disgust: quadratic, fear: linear, sadness: linear, joy: linear, neutral: linear). (**B**) Graphic of the linear mixed model outputs using duration and emotion presented (model with four gates). Shown is the unbiased estimated percentage of correct recognition of the emotion as expressed over time (divided into four gates representing the percentage of the full stimulus presented, e.g., 25%, 50%, 75%, and 100%). The values were computed with a linear mixed model that evaluated the percentage of correctness using the emotion expressed, the gate duration, and their interaction as predictors. The error bars represent the confidence interval at 95%.

Three results indicated the importance of the amount of information given through the gating paradigm in order to accurately recognize emotions. First, with the gates created (see Method section), we computed the confusion matrix for every emotion through the fine-grained gates: 10%, 20%, and so on (S2 Table). The matrix showed the distribution of emotion chosen at every gate for every emotion expressed in the stimuli. This distribution changed over time and became more specific to the emotion presented at later gates. It was also important to notice that the total duration of the stimuli used in this study varied greatly across emotion. On average, affect burst in our study varied in length from 825ms for fearful stimuli to 2325ms for happy stimuli, resulting in different mean gate durations (S5 Table). Secondly, from this confusion matrix, we were able to derive the unbiased scores (Hu scores) for every emotion at every gate (Fig 1A). This score highlighted that some emotions, e.g. anger, disgust, and fear, are recognized more rapidly and accurately than the rest of the presented emotions.

We also computed a model estimating the Hu scores at cruder gates (25%-50%-75%-100%) using the duration of the stimuli as fixed effect and the participants as random intercepts effect. When this model was compared statistically with a model that took into account only the random effects of the participants, the model encompassing the duration effect was significantly better, *χ*^2^(6, *N* = 1, 920) = 79.99, *p* < 0.001, $R_{m}^{2}=0.04,R_{c}^{2}=0.10$. The Akaike information criterion (AIC) and Bayesian information criterion (BIC) showed a better relative quality of the statistical model for encompassing gate durations (*AIC*_*Dur*_ = 331, *AIC*_*rand*_ = 405; *BIC*_*Dur*_ = 364, *BIC*_*rand*_ = 421). This third result show the importance of duration on the unbiased scores.

Finally, we created another model, adding the influence of the emotion presented as the main effect and later also as an interaction effect in the model with duration. When comparing to a model without this interaction, the final model highlighted the significant impact of the interaction between the emotion presented and the duration of the stimuli on the unbiased emotion recognition *χ*^2^(26, *N* = 1, 920) = 124.14, *p* < 0.001, $R_{m}^{2}=0.38,R_{c}^{2}=0.46$, *AIC*_*EmoxDur*_ = −580, *AIC*_*Emox*+*Dur*_ = −485; *BIC*_*EmoxDur*_ = −435, *BIC*_*Emox*+*Dur*_ = −424. For completeness, a similar model using the raw emotion recognition and a model estimating the raw emotion ratings using every 50ms gates were also computed (S2 and S3 Figs).

Our final model estimated the unbiased accuracy of recognition of the six different emotions presented (anger, disgust, fear, joy, sadness, and neutral) over time using affect bursts (Fig 1B). Each emotion was recognized at a specific rate over time and all emotions except neutral were better recognized at later gates. Anger, disgust, and fear were better recognized overall with the best accuracy occurring at a later gate. Neutral was the only emotion for which recognition accuracy decreased over time. Finally, at the last gate, when stimuli were presented at their full duration, emotions such as anger, disgust, and fear were significantly better recognized than sadness, joy, and neutral (Table 2). In terms of best recognition, emotions can be rearranged as follows: *anger* > *disgust* > *fear* > *joy*/*sadness*, *joy*/*sadness* > *neutral*.

**Table 2:** Contrasts comparing Hu scores at last gate between emotions.

	Disgust	Fear	Joy	Neutral	Sadness
Anger	*χ*^2^(1, *N* = 160) = 56.03, *p* < 0.001	*χ*^2^(1, *N* = 160) = 95.29, *p* < 0.001	*χ*^2^(1, *N* = 160) = 203.26, *p* < 0.001	*χ*^2^(1, *N* = 160) = 317.53, *p* < 0.001	*χ*^2^(1, *N* = 160) = 183.31, *p* < 0.001
Disgust		*χ*^22^(1, *N* = 160) = 5.18, *p* < 0.001	*χ*^2^(1, *N* = 160) = 45.85, *p* < 0.001	*χ*^2^(1, *N* = 160) = 106.79, *p* < 0.001	*χ*^2^(1, *N* = 160) = 36.65, *p* < 0.001
Fear			*χ*^2^(1, *N* = 160) = 20.2, *p* < 0.001	*χ*^2^(1, *N* = 160) = 64.92, *p* < 0.001	*χ*^2^(1, *N* = 160) = 14.26, *p* < 0.001
Joy				*χ*^2^(1, *N* = 160) = 12.69, *p* < 0.001	*ns*
Neutral					*χ*^2^(1, *N* = 160) = 18.32, *p* < 0.001

All significance level are FDR corrected (corrected for multiple comparisons using a false discovery rate correction).

### Recognition certainty

Using a similar procedure, we demonstrated that the duration of the gates significantly influenced the participants’ confidence level, *χ*^2^(11, *N* = 8, 400) = 315.24, *p* < 0.001, $R_{m}^{2}=0.06$, $R_{c}^{2}=0.36$.

**Fig 2:**
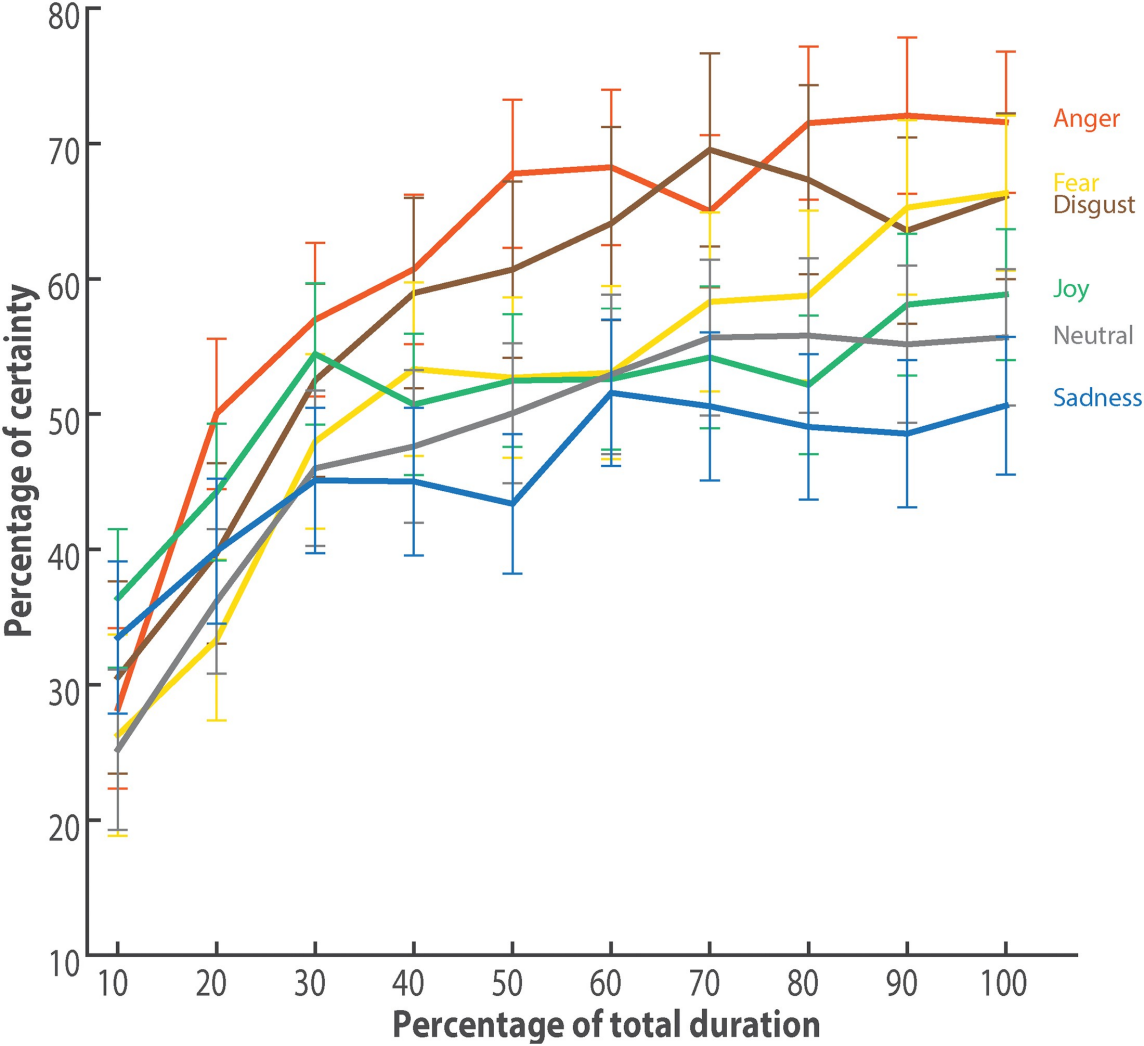
Certainty over time. Graphic of the generalized linear mixed model outputs duration and emotion presented. Shown is the estimated self-confidence percentage for each emotion over time. The values were computed with a generalized linear mixed model that evaluated the percentage of confidence using the emotion expressed, the gate duration, and their interaction as predictors. The error bars represent the confidence interval at 95%.

A LMM was computed to estimate the certainty level at each gate (10%-20%-..-100%). The most complete model used as a fixed effect the emotion expressed and the duration, as well as their interaction. The interaction between the emotion expressed and the duration significantly improved the model, *χ*^2^(61, *N* = 8,400) = 172.91, *p* < 0.001, $R_{m}^{2}=0.1$, $R_{c}^{2}=0.4$ (Fig 2), compared to a model with no interaction.

We can notice here that participants were significantly less confident at shorter gates than longer ones (*χ*^2^(1, *N* = 8400) = 189.01, *p* < 0.001). While there is no statistical different between emotions at those shorter gates, a similar pattern as for emotion recognition can be observed at the last gate. In terms of strongest certainty in ratings, emotions can be arranged as follows: *anger* > *fear*, *disgust* > *joy*, *neutral* > *sadness*.

### Linear and nonlinear recognition curves

To compare the different recognition curve shapes, we used two different methods. The first one computed polynomial fits on the unbiased hit rate over the 10% gates (Fig 1A). The RMSE and adjusted squared were used as means of determining the best corresponding fit. The second method estimated orthogonal polynomial contrasts on the last linear mixed model using the 25% gates (Fig 1A and 1B). Both methods highlighted the need for higher polynomial fit for both anger and disgust, respectively third and second order, whereas the rest of the emotions are well defined by linear relationships (Table 3).

**Table 3:** Comparison between polynomial fits and orthogonal polynomial contrasts for each emotion curve.

Polynomial contrast (Root mean squared error, adjusted R squared) [10 gates (10%-20%-..-100%) dataset] Orthogonal Polynomial Contrast (FDR corrected) [on 4 gates (25%-50%-75%-100%) dataset]		Equation (duration in seconds)
Anger	Chi-squared test *χ*^2^(1, *N* = 320)	*anger* = 1.694*dur*^3^ − 3.955*dur*^2^ + 2.946*dur* − 0.044
Linear	*RMSE* = 0.125, *R*^2^ = 0.514(*χ*^2^ = 68.12, *p* < 0.001)	
Quadratic	*RMSE* = 0.106, *R*^2^ = 0.654(*χ*^2^ = 10.10, *p* = 0.002)	
Cubic	*RMSE* = 0.080, *R*^2^ = 0.802(*χ*^2^ = 15.91, *p* < 0.001)	
Disgust	Chi-squared test *χ*^2^(1, *N* = 320)	*disgust* = −0.942*dur*^2^ + 1.251*dur* + 0.077
Linear	*RMSE* = 0.073, *R*^2^ = 0.671(*χ*^2^ = 36.55, *p* < 0.001)	
Quadratic	*RMSE* = 0.054, *R*^2^ = 0.8659(*χ*^2^ = 11.26, *p* = 0.001)	
Cubic	*RMSE* = 0.051, *R*^2^ = 0.838, *ns*	
Fear	Chi-squared test *χ*^2^(1, *N* = 320)	*fear* = 0.224*dur* + 0.089
Linear	*RMSE* = 0.043, *R*^2^ = 0.845(*χ*^2^ = 52.18, *p* < 0.001)	
Quadratic	*RMSE* = 0.041, *R*^2^ = 0.859, *ns*	
Cubic	*RMSE* = 0.044, *R*^2^ = 0.838, *ns*	
Joy	Chi-squared test *χ*^2^(1, *N* = 320)	*joy* = 0.093*dur* − 0.008
Linear	*RMSE* = 0.022, *R*^2^ = 0.902(*χ*^2^ = 31.39, *p* < 0.001)	
Quadratic	*RMSE* = 0.016, *R*^2^ = 0.946, *ns*	
Cubic	*RMSE* = 0.017, *R*^2^ = 0.942, *ns*	
Neutral	Chi-squared test *χ*^2^(1, *N* = 320)	*neutral* = −0.131*dur* + 0.194
Linear	*RMSE* = 0.020, *R*^2^ = 0.844(*χ*^2^ = 14.04, *p* < 0.001)	
Quadratic	*RMSE* = 0.015, *R*^2^ = 0.915, *ns*	
Cubic	*RMSE* = 0.014, *R*^2^ = 0.921, *ns*	
Sadness	Chi-squared test *χ*^2^(1, *N* = 320)	*sadness* = 0.099*dur* + 0.084
Linear	*RMSE* = 0.028, *R*^2^ = 0.802(*χ*^2^ = 17.28, *p* < 0.001)	
Quadratic	*RMSE* = 0.018, *R*^2^ = 0.916, *ns*	
Cubic	*RMSE* = 0.019, *R*^2^ = 0.904, *ns*	

The polynomial fit are computed on the Hu scores computed from the confusion matrix. These fit are charasterised by the root mean squared error (RMSE) and the adjusted R-squared (*R*^2^). The orthogonal polynomial contrasts are computed on the linear mixed models encompassing the interaction of emotion presented and duration as a fixed effect (4 gates). The orthogonal polynomial contrasts are compared using chi-squared test and p-values. FDR corrected = corrected for multiple comparisons using a false discovery rate correction.

With the orthogonal polynomial contrasts computed, we compared the slopes for every emotion at every gate (Fig 1B). The slopes of the recognition curves varied from gate to gate. The neutral slope on the first gate was significantly different from that for the other emotions (Table 4). At lower gates, anger also had a significantly steeper slope than the other emotions except disgust. The differences between the slopes at later gates were non-significant.

**Table 4:** Comparison of the slopes of the different emotion recognition curves between the first and second gate.

Chi-squared test comparing slopes between emotions (FDR corrected)					
	Disgust	Fear	Joy	Neutral	Sadness
25%–50%					
Anger	*ns*	*χ*^2^(1, *N* = 160) = 7.47, *p* = 0.040	*χ*^2^(1, *N* = 160) = 21.28, *p* < 0.001	*χ*^2^(1, *N* = 160) = 44.04, *p* < 0.001	*χ*^2^(1, *N* = 160) = 13.38, *p* = 0.002
Disgust		*ns*	*ns*	*χ*^2^(1, *N* = 160) = 19.55, *p* < 0.001	*ns*
Fear			*ns*	*χ*^2^(1, *N* = 160) = 15.26, *p* = 0.001	*ns*
Joy				*ns*	*ns*
Neutral					*χ*^2^(1, *N* = 160) = 8.87, *p* = 0.021

All the comparisons at later gates were non-significant. Chi-squared tests comparing slopes between different emotions for the model using four gates. All p-values are corrected for multiple comparisons using a false discovery rate correction.

### Impact of acoustic parameters

**Fig 3:**
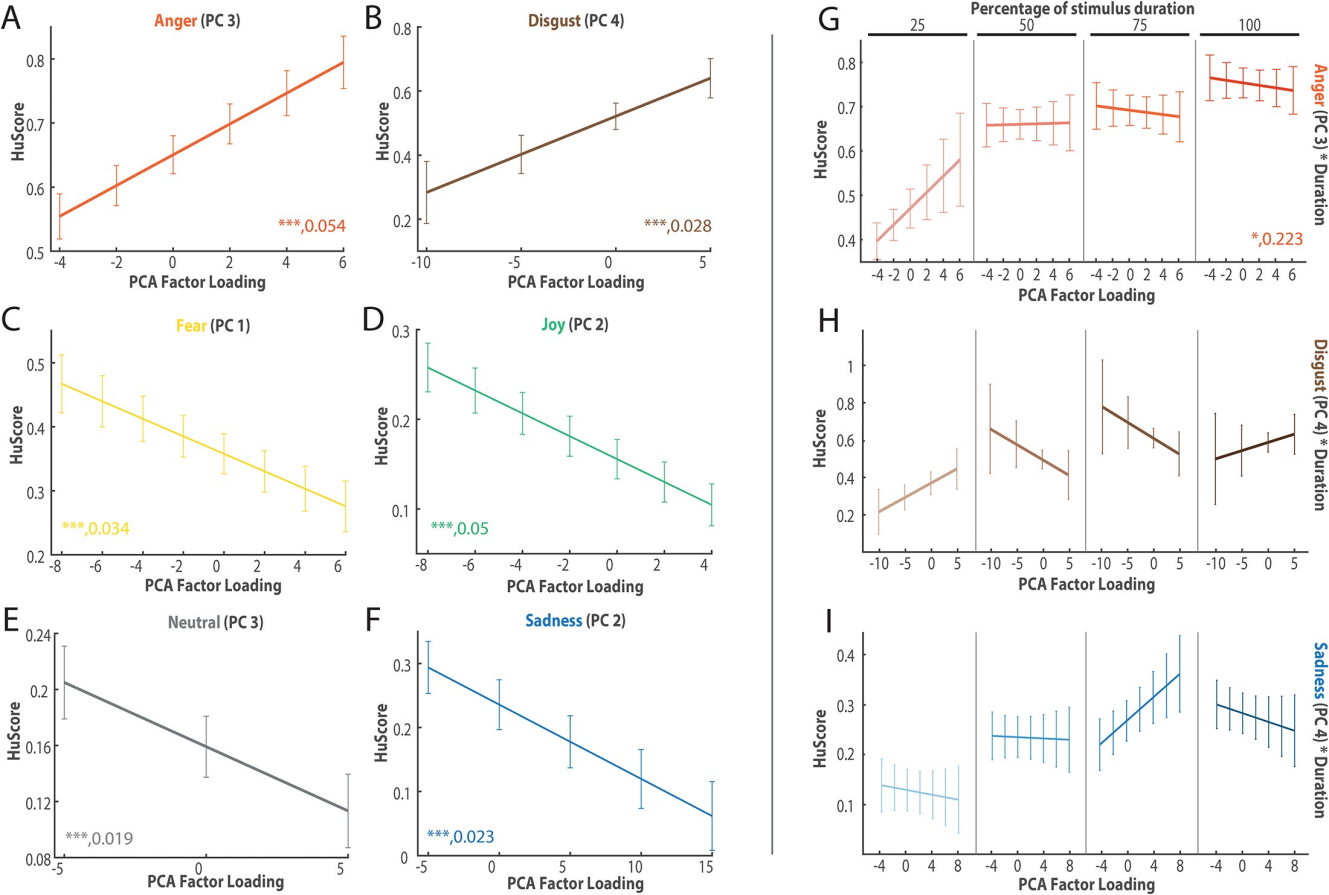
Impact of acoustic features on recognition across and over time. Graphic of the generalized linear mixed model outputs using the principal component (PC) scores computed independently for every emotion (**A,B,C,D,E,F**) and the interaction with the duration of the stimulus (**G,H,I**). Shown is the unbiased estimated percentage of correctness for each emotion. The values were computed separately for each emotion with a linear mixed model that evaluated the unbiased percentage of correctness using PC scores. The error bars represent the confidence interval at 95%. (**A**) Anger PC3, (**B**) Disgust PC4, (**C**) Fear PC1, (**D**) Joy PC2 (**E**) Neutral PC3, (**F**) Sadness PC2, (**G**) third PC and duration for anger, (**H**) fourth PC and duration for disgust, (**I**) fourth PC and duration for sadness. The numbers associated with the data represent the significance level (FDR-corrected) as well as the statistical power shown in S6 Table. *: *p* < 0.05, **: *p* < 0.01, ***: *p* < 0.001.

A principal component analysis was computed using 42 acoustic features from the Geneva Minimal Acoustic Set [28] extracted for each stimulus (S6 Table). We took the decision of computing the PCs respective to each emotion since they exhibited different patterns of acoustic cues in previous published articles [2, 35]. We extracted four PCs for each emotion based on the examination of the scree plots and cumulative eigenvalue for each PC (S5 Fig). Each PC corresponds to its own set of acoustic features depending on the emotion portrayed in the stimuli used to compute it. We create LMMs to estimate the unbiased accuracy of recognition with each of the four PC for each specific emotion separately (Fig 3A, 3B, 3C, 3D, 3E and 3F). For example, we estimated the Hu scores linked only to angry stimuli based on principal components extracted from those angry stimuli. We also examined models the influence of each PC at each gate, incorporating information about the duration of the stimuli(Fig 3G, 3H and 3I).

The accuracy of recognition of specific emotions was significantly modulated by some of their specific PCs (S6 Table). The recognition of anger was the most significantly impacted by the values of its PC3; disgust by PC4; fear by PC1; joy by PC2; neutral by PC3; and finally sadness by PC2. In multiple cases, the variance in the unbiased recognition of each emotion could be decently explained by the use of their respective PCs. Specifically, recognition of anger, joy and fear could be decently well described respectively by their PC3, PC2, PC1 ($R_{m}^{2}>0.03$). The third principal component for anger was composed of loudness and spectral characteristics such as standard deviation for the main fundamental frequency (F0) and mean amplitude for the second and the third fundamental frequency. As the values of these features increased, recognition was improved. The second principal component for joy was composed of negative weights for loudness of rising slopes, standard deviation of loudness, and mean and standard deviation of F0. Taking the negative weights into account, as the value for those characteristics decreased, the unbiased hit rate decreased as well. Finally, the first principal component for fear encompassed the Hammarberg index and slope, and was negatively linked to loudness. Recognition of fearful affect bursts was less accurate when the Hammarberg index increased and the loudness decreased.

When computing a model integrating the interaction of the PC and the duration of the stimulus, only anger, disgust, and sadness showed significant differences compared to models using only the main effects (S6 Table). These models highlighted the impact of their respective PC 3, 4, and 4 on the different gates (Fig 3G, 3H and 3I). PC4 for anger was inversely associated with the the formant F1, F2, and F3 while PC4 for sadness was mainly composed of loudness slope and inversely associated with jitter (associated with the quality of the vocal excitation). The most noticeable impact of respective PC happened at different gates for each emotion. For anger, it happened at first gate with an increase of the unbiased recognition while a bit later for disgust (second and third gate) highlighting a decrease of the Hu scores. The impact of the fourth PC for sadness was most noticeable at the third gate and was associated with an increase of the Hu scores.

## Discussion

Overall, this study emphasized that accuracy in recognizing emotional prosody increases over time in gating paradigms using affect bursts. There are indeed distinct emotion-specific shape functions for the recognition curves. Moreover, acoustic features play a role in distinguishing between different emotions.

### Recognition of emotion

The main goal of this paper was to characterize how the recognition of discrete vocal emotion in affect bursts evolves over time through a powerful method of analysis, the Linear Mixed Models, using a gating paradigm. We aimed to compare the different recognition patterns for different emotions. The results demonstrate that the recognition of emotions increased incrementally over the course of a stimulus, with the exception of neutral stimuli. For non-neutral emotions, the recognition rate increased and confidence ratings improved during longer utterances. This pattern implies that listening to longer portions of an utterance facilitates processes of explicit recognition and the ability to decode emotional prosody with ease and more confidence [17, 36, 37].

The analysis of responses given for the last gate (when stimuli were presented at their full length) corroborates results found in forced-choice experiments where full utterances are presented to the participants. Certain emotions can be recognized significantly better than others from the voice when evaluated in forced-choice experiments [4, 38–41]. In the present experiment, all non-neutral emotions were also recognized at full duration at an above-chance level and some emotions—anger, disgust, and fear—were recognized significantly better than emotions such as neutral, sadness, and joy.

Anger, disgust, and fear were detected faster and more accurately than other vocal expressions of basic emotion. This is probably because negative emotions convey information about threats, aggression, and danger. Negative and arousing emotions might contain more information, potentially to allow individuals to react quickly and appropriately to undesirable stimuli [42, 43]. Neural systems, action tendencies, and cognitive responses have now been well defined in association with aversive or threatening stimuli, such as facial and vocal expressions of fear and anger [9–11]. Many researchers report that fear is usually recognized faster and more accurately than other vocal expression of basic emotions [44, 45]. Fearful voices exhibited a higher mean pitch and faster speech rate than did other emotions [2, 39], which led to better distinct recognition on the perceptual level of analysis. Noteworthy is the fact that faster speech rate would lead to shorter excerpts in the case of fearful voices. In our dataset, fearful bursts were on average the shortest recordings alongside anger, disgust, and neutral. This fact suggests that actors portrayed these emotions as really short bursts and it is to be accepted that they would be expressed similarly in real life. Consequently, it is possible than the human emotion recognition system is biased towards associating shorter excerpts with these four emotions, namely anger, disgust, fear, and neutral.

Affect bursts portraying disgust are efficiently recognized. While multiple articles report results that reflect a less accurate and slower ability to recognize vocal attributes of disgust during speech processing [2, 40, 46], studies about affect burst reported that stimuli portraying disgust were associated with the best recognition accuracy [24, 26]. One possible explanation is that disgust is usually expressed by the means of affect bursts, and is most unlikely to be expressed through manner of speaking compared to the other emotions [2]. The recognition of this specific usual sound *‘aaargh’* appears to be extremely fast for participants because of the uniqueness of its usage.

Sadness and joy, in comparison to fear, anger, and disgust, were poorly recognized during the first gates and even during the presentation of the full-length utterances. Although the correctness of the ratings increased over the course of an utterance, the scores obtained with the full duration remained lower than for emotions such as anger, fear, and disgust. In accordance with this finding, multiple studies showed that joy tends to be harder to detect in speech prosody than most basic emotions [44, 45, 47, 48]. Furthermore, the broad term ‘joy’ used in this study might not have reflected the precise content of the recordings. It was shown that listeners could differentiate between multiple shades of positive emotions beyond happiness, namely achievement, amusement, contentment, sensual pleasure, relief [25]. Offering more options to choose from might have yield different results and improved the general accuracy. Finally, the lack of advantages for accurately recognizing happy expressions is predicted by the literature, although our data show that these difficulties extend to a slower recognition speed [17]. Contrary to what is reported in the literature [38], here sadness had an overall poor recognition rate. The onomatopoeia for sadness sounds similar to moaning, which was confused with neutral or even joyful stimuli by our participants. Contrarily to disgust, sadness might be better conveyed through language and the use of affect bursts might feel unusual and unnatural for listeners.

The accuracy of recognition of neutral expressions was the only one that decreased over time. A recent study concluded that there may be an advantage for recognizing neutral, rather than emotional, content during emotional prosody processing [17]. However, in this particular case, responses for neutral utterances showed a much higher rate of false positive recognitions. These results were linked to the paradigm used and the forced-choice condition. At lower gates, when information was lacking, participants tended to select neutral more often than other emotions, resulting in a higher false alarm rate. By forcing participants to choose between the six emotions, neutral was selected more often as a default value, creating a virtually higher percentage of correctness for neutral utterances at lower gates. This should be corrected by giving clear instructions to participants or allowing them to select an option other than the basic emotion when they are unsure. The deterioration of the recognition of neutral stimuli in the unbiased model might be linked to the design of the experiment. The fact that short and long stimuli were presented in random order might have induced the participants to balance the different emotions chosen. Although participants mainly selected neutral in the shorter stimuli, the longer neutral utterances were less likely to be selected as neutral. Moreover, the affect burst */aaa/* seems not to be the best way to express neutral emotion because it is usually confused with sadness.

Our findings show similar pattern of accuracy as the GEMEP database from which our stimuli have been selected [29]. In the GEMEP database, hot anger audio stimuli were recognized with 76% accuracy, disgust 10%, panic fear 71%, sadness 46%, joy 40%, and neutral (interest) 31%. With the exception of disgust, all emotions follow the same order in terms of recognition accuracy. This is due to the fact that the GEMEP database is composed of affect bursts as well as two types of phonetically constructed pseudo-sentences. The poor accuracy in the GEMEP database reflects the likelihood that disgust is poorly recognized and portrayed by long utterances of language and is better mimicked using affect bursts [2].

Compared to a previous study where identification points were calculated, e.g fear (*M* = 517*ms*), sadness (*M* = 576*ms*) [16], our study differs in two ways. The presentation of stimuli was pseudo-randomized in such a way that participants presented with a specific stimulus at short gates might never listen to a longer gate of the same stimulus. It was thus impossible to pinpoint an exact identification point in time that corresponded to the moment when a participant correctly recognized the emotion presented and never changed his or her mind. The gating paradigm defined by Grosjean (1980) suggested a duration-blocked format, which would always start with the shortest gates and end with the presentation of a block of all the full-duration stimuli [12]. This limitation in our design prevented us from calculating a precise timing. Secondly, affect bursts are defined as “very brief, discrete, non-verbal expression of affects” [22]. Their very brief nature should be linked to faster recognition than elaborate semantic utterances making it more difficult to compare. Furthermore, by creating gates in 50 ms intervals, we made participants rate shorter stimuli than most gating studies [16, 17].

### Shape functions

One of the main findings revealed by this data is that the recognition of basic emotions expressed by emotional prosody is linked to a distinct shape functions. Recognition accuracy evolved through our gating paradigm and demonstrated that there were distinctively marked emotion-specific polynomial functions (e.g., linear, quadratic). The accumulating acoustic evidence that unfolded at every gate seems to shape the recognition of basic emotions encoded in the voice. For example, the recognition curves for anger and disgust in particular showed a large increase across the first gates. Their respective recognition curves are better described using nonlinear regressions. The orthogonal polynomial as well as the RMSE of the polynomial fit contrasts showed the use of second to third degrees for such emotions. As stated earlier, these emotions induce faster responses in individuals, potentially allowing them to react quickly and appropriately to undesirable stimuli [42, 43]. Emotions such as fear, sadness, joy, and neutral are better described by a linear relationship between the gates and by recognition accuracy. The amount of information contained in the respective affect bursts improves linearly the recognition accuracy over time.

The shape functions of different basic emotions over time is, to the best of our knowledge, formally described for the first time in this study. This provides an interesting mathematical definition of the changes of recognition accuracy over time. These mathematical equations allow for predictions and precise comparisons. They favor a more reliable and accurate exportation of our results than mere graphical observations. These results are completed here by the comparison between the slopes from the recognition curves for each emotion. With the analysis, we reinforced the finding that anger showed a particularly high increase in recognition accuracy at the first gates, in line with the fast reaction required when someone faces threats or undesirable stimuli.

### Acoustic features

The specific emotions examined in this study—anger, disgust, fear, sadness, and joy—are all believed to have evolved as unique signal functions in human communication. Consequently, they are thought to be each encoded and decoded in a unique fashion [1, 49]. The theoretical framework behind those specific signals hypothesized that affective states affects the tension in the striate musculature and change the autonomic arousal resulting in affecting both voice and speech production [50]. The correct recognition of vocal emotion relies on sharing the same knowledge about what a vocal emotion sounds like. In this study, a well-documented set of acoustic features were used to define characteristics linked to the sound wave of each stimulus [28]. The Geneva Minimal Set of Acoustic Parameters extracts parameters related to frequency (associated with pitch), energy/amplitude (linked to loudness), as well as spectral parameter (e.g. harmonic differences, hammarberg index) on the basis of previous work [51–53].

Groups of acoustic features emerged for each emotion by using a PCA. McGilloway et al. (2000) thought it was reasonable to believe that the decoding of acoustic features is nonlinear (e.g., the relevance of one feature is conditional on others) [54]. The different relationships between the recognition of prosody and the related acoustic features shown in these results suggest it once more. Since emotions are also defined by sets of specific acoustic features [2, 35], the focus of this work lies in the analysis of PCs for one emotion at a time.

Anger was best recognized when the model incorporated the third PC representing the normalized standard deviation for the fundamental frequency (f0, associated with pitch) and jitter (associated with the quality of the vocal excitation). Anger is defined in multiple studies as increased f0 mean, variance, and range [53, 55, 56]. Even though the third PC was also described by a few loudness parameters, most parameters were associated with frequency and spectrum, as previously established by the literature in the case of non-verbal communication [52]. The data here are in line with this definition since better recognition is achieved when these features have higher values within the PC, resulting in a higher score.

The joyful bursts in our study were represented by the classic */a-a-a-a/* or *“ahahahah,”* which is a succession of the /a/ sound each time followed by a drop in loudness. The recognition of joyful bursts was mainly characterized by its second PC. It was inversely related to parameters such as pitch mean and standard deviation and loudness standard deviation and range. Taken together with the negative slope describing the Hu scores associated with this PC, higher values for these parameters meant a better accuracy. Joy and happiness have been characterized multiple times by a higher mean pitch and higher pitch range [57–59]. The best component from the PCA analysis for joy was also characterized with spectral components such as the slope of the logarithmic power spectrum within the two given bands. Parameters related to amplitude and spectrum are both reported to be linked to pleasurable non-verbal communication [52].

Fear is the last emotion for which acoustic parameters significantly helped recognition. The first PC was characterized by positive loadings for spectral parameter such as the slope of the logarithmic power spectrum within the two given bands and the Hammarberg index mean. Negative loadings were associated with loudness mean, range, hand the standard deviation of Hammarberg index. This index is defined as ratio of the strongest energy peak in the 0-2 kHz region to the strongest peak in the 2–5 kHz region [60]. Lower Hammarberg was associated with more accurate recognition of fearful stimuli in our study. Combined with the slope of the logarithmic power spectrum, this provides evidence that bigger differences in spectral power when comparing higher frequency bands to lower frequency bands boosts recognition accuracy. Spectral components were similarly highlighted for fearful bursts [52]. Louder fearful bursts were also better recognized. Since our recordings were very similar to shouts or screams, loudness was expected to play a role.

Finally, we observed a differential impact of the principal components for anger, disgust, and sadness depending on the duration of the stimulus. For anger, the strong influence of PC values at the lowest gate dissipated at the longer gates. This would assume the ability to decode quickly acoustic features related to anger in order to prepare the individual for a fight or flight response. When the amount of information available is low, the human brain bases its assumptions presumably on basic acoustic features. With longer stimuli and the amount of information available increasing, the work done at the lowest gate is further refined but the supplementary amount of information has less of an impact. For disgust, this strong influence of PC values happened at longer gates while extending even further for sadness. Extending this logic, we could see that the impact of the principal component in predicting the unbiased percentage of correctness depended on the amount of information available. While only a short amount of information is necessary to compute the features helpful to decode anger, longer utterances were required for disgust and sadness. In the end, when this required amount of information was presented, our results showed that acoustic features associated helped improving recognition.

## Conclusion

With the help of linear mixed models and a gating paradigm, this study highlighted the importance of studying the time course at which vocal expressions of emotion unfold. Specifically, this line of research focused on affect bursts as a mean of communicating accurately emotions. We observed that some emotions, namely anger, disgust, and fear were recognized faster and more accurately than joy, sadness, and neutral. We reported non-linear relationships between the duration of the stimuli presented and the accuracy of the response for anger and disgust. Finally, we explored acoustics features associated with affect bursts such as loudness, pitch, and the spectrum of frequencies, highlighting both their importance in improving recognition and their impact over time. More specifically, we observed that the recognition of angry stimuli was improved with higher values for the standard deviation of the fundamental frequency and jitter while joyful stimuli were better recognized by higher pitch and greater loudness range. Furthermore, the impact of acoustic features related to anger, disgust, and sadness varied over time. Taken together, the results demonstrate the potential of using a gating paradigm with affect burst and deepen the basic knowledge about the time course of emotion recognition.

## Supporting information

S1 Appendix Raw data. Data collected during the experiment. This corresponds to the responses of 80 participants over 8,400 stimuli.
(CSV)

S2 Appendix Impact of gender on emotion recognition. Computation of a model encompassing the gender of our participants as fixed effect. This model highlights the lack of impact of gender on the accuracy of recognition of affect bursts in our experimental set-up.
(PDF)

S1 Fig Model emotionpresented x scales. Graphic of the generalized linear mixed model outputs using scales and emotion presented. Shown is the estimated percentage of emotion responses chosen per category of emotional vocalization. The values were computed with a general linear mixed model that evaluated the percentage of emotion chosen with the emotion chosen, the emotion expressed, and their interaction as predictors. Each line represents all stimuli of a particular emotion. The error bars represent the confidence interval at 95%.
(PDF)

S2 Fig Biased recognition of emotions over time. Graphic of the generalized linear mixed model outputs using duration and emotion presented (model with 10 gates). Shown is the estimated percentage of correct recognition of the emotion expressed over time (divided into 10 gates representing the percentage of the full stimulus presented, e.g., 10%, 20%…90%, and 100%). The values were computed with a generalized linear mixed model that evaluated the percentage of correctness using the emotion expressed, the gate duration, and their interaction as predictors. The error bars represent the confidence interval at 95%. The chance level represents the percentage of choosing one of the seven emotions by chance. Continuous thinner lines represent the best polynomial fit based on the orthogonal polynomial contrasts performed in Table 3 (anger: cubic, disgust: quadratic, fear: quadratic, sadness: quadratic, joy: linear, neutral: quadratic). The recognition of the neutral emotion at the early gates revealed that judged neutrality was on average selected by the participants more often than were the other emotions, causing a clear bias (at the first 25% of total duration presented, participants selected the following as the preferred response: neutral: 648, anger: 208, surprise: 279, fear: 386, joy: 134, sadness: 172, disgust: 124). The confusion matrix also showed this bias with a more important proportion of responses for neutral than for the other choices (S2 Table). To control for this bias, we calculated the unbiased rating (Hu Scores) for each gate and each emotion (Fig 1).
(PDF)

S3 Fig Rating over time (50ms increments). Estimation of the input value for specific emotions for every 50ms increments. We used separate emotions to estimate the input continuous value (0-100) using linear mixed models.
(PDF)

S4 Fig Recognition unfolding of stimuli recognized at the same rate at full-length. Model using only a sub-set of stimuli from different emotions that are matched for recognition at full-length. This was computed in order to rule out the possibility that differences in recognition at earlier time-points merely reflects the ease of recognition of the tokens of different emotions in this stimulus set. Recognition curves reveal a similar pattern with anger, disgust, and fear being recognized more accurately at earlier gates, while sadness and joy are less recognized at earlier gates. Neutral is not depicted here due to its biased nature in our study.
(PDF)

S5 Fig Scree plots PCA. Scree plot for the principal component analysis (PCA) for each emotion separately.
(PDF)

S1 Table Mean and range of excerpts duration. Mean and Range of Total Duration and Number of Subdivisions for Each Emotion Separately.
(PDF)

S2 Table Confusion matrix. Confusion matrix between the emotion presented and the emotion chosen by participants at different points in time.
(PDF)

S3 Table PCA & acoustic features. Loading Factors of the Principal Component Analysis for the Six Different Emotions Taken Individually.
(PDF)

S4 Table Contrast emotionpresented x scales. Contrast computations between a specific scale when the corresponding emotion is presented and all other scales.
(PDF)

S5 Table Mean and standard deviation of the gate durations. Mean and Standard Deviation of the Gate Durations for Each Emotion Separately.
(PDF)

S6 Table PCA models for each emotions and over time. Comparisons between general linear mixed models using the PCs computed with the principal component analysis on each emotion separately.
(PDF)
