# Supplementary material for: Unfolding and dynamics of affect bursts decoding in humans
Source: PLoS One. 2018 Oct 30;13(10):e0206216. doi: 10.1371/journal.pone.0206216 (PMC6207317; doi:10.1371/journal.pone.0206216)
Supplement: S2 Appendix — Computation of a model encompassing the gender of our participants as fixed effect. This model highlights the lack of impact of gender on the accuracy of recognition of affect bursts in our experimental set-up. (PDF) [file pone.0206216.s002.pdf]

Impact of gender on emotion recognition.

There was a significant unbalance in the participants' gender for this experiment with 62 females for 18 males. In order to control for the effect of gender on emotion recognition, we ran two models. The first model estimated the Hu score based on gender only as fixed effect and the participants as random effect. When comparing this model to an empty model, we could acknowledge a significant improvement: $\chi 2(4, N = 1, 920) = 7.206$, $p = 0.007$, $R_m^2 = 0.006$, $R_c^2 = 0.032$, $AIC_{Gender} = 790.5$, $AIC_1 = 795.7$; $BIC_{Gender} = 812.7$, $BIC_1 = 812.4$. Despite the significance of the chi-squared test, we observed that a model encompassing gender as fixed effect does not seem to improve the Bayesian Information Criterion and is associated with almost no statistical power. The second model we computed estimated the Hu scores with the interaction between gender and the emotion presented. This second model failed to be significantly different from a model with only the main effect of both gender and emotion. The interaction between gender and the emotion presented does not seem to contribute to improve the estimation of the Hu scores. We concluded that gender has little to no impact on our results despite the unbalance in our pool of participants
