# Supplementary material for: Unfolding and dynamics of affect bursts decoding in humans
Source: PLoS One. 2018 Oct 30;13(10):e0206216. doi: 10.1371/journal.pone.0206216 (PMC6207317; doi:10.1371/journal.pone.0206216)

Graphic of the generalized linear mixed model outputs using scales and emotion presented. Shown is the estimated percentage of emotion responses chosen per category of emotional vocalization. The values were computed with a general linear mixed model that evaluated the percentage of emotion chosen with the emotion chosen, the emotion expressed, and their interaction as predictors. Each line represents all stimuli of a particular emotion. The error bars represent the confidence interval at 95%.

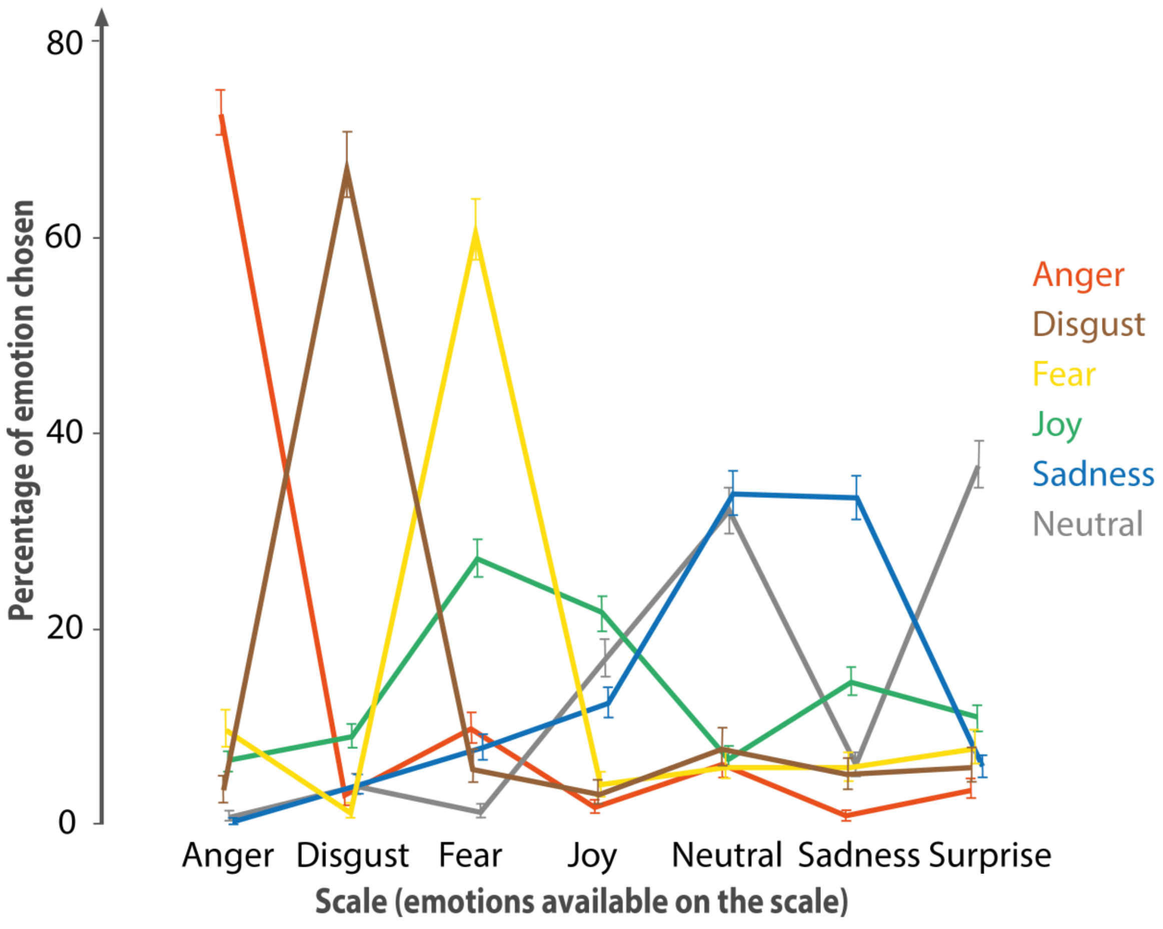

Supplement: S1 Fig — Graphic of the generalized linear mixed model outputs using scales and emotion presented. Shown is the estimated percentage of emotion responses chosen per category of emotional vocalization. The values were computed with a general linear mixed model that evaluated the percentage of emotion chosen with the emotion chosen, the emotion expressed, and their interaction as predictors. Each line represents all stimuli of a particular emotion. The error bars represent the confidence interval at 95%. (PDF) [file pone.0206216.s003.pdf]
