# Supplementary material for: Unfolding and dynamics of affect bursts decoding in humans
Source: PLoS One. 2018 Oct 30;13(10):e0206216. doi: 10.1371/journal.pone.0206216 (PMC6207317; doi:10.1371/journal.pone.0206216)

Graphic of the generalized linear mixed model outputs using duration and emotion presented (model with 10 gates). Shown is the estimated percentage of correct recognition of the emotion expressed over time (divided into 10 gates representing the percentage of the full stimulus presented, e.g., 10%, 20%…90%, and 100%). The values were computed with a generalized linear mixed model that evaluated the percentage of correctness using the emotion expressed, the gate duration, and their interaction as predictors. The error bars represent the confidence interval at 95%. The chance level represents the percentage of choosing one of the seven emotions by chance. Continuous thinner lines represent the best polynomial fit based on the orthogonal polynomial contrasts performed in Table 3 (anger: cubic, disgust: quadratic, fear: quadratic, sadness: quadratic, joy: linear, neutral: quadratic). The recognition of the neutral emotion at the early gates revealed that judged neutrality was on average selected by the participants more often than were the other emotions, causing a clear bias (at the first 25% of total duration presented, participants selected the following as the preferred response: neutral: 648, anger: 208, surprise: 279, fear: 386, joy: 134, sadness: 172, disgust: 124). The confusion matrix also showed this bias with a more important proportion of responses for neutral than for the other choices (Appendix D). To control for this bias, we calculated the unbiased rating (Hu Score) for each gate and each emotion (Figure 1)

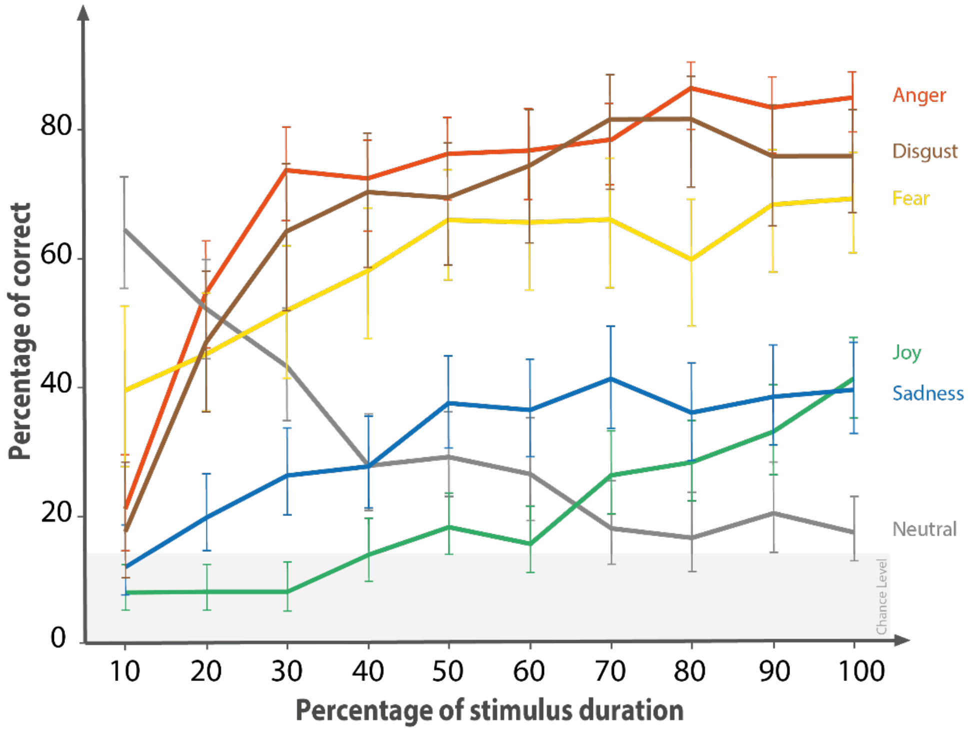

Supplement: S2 Fig — Graphic of the generalized linear mixed model outputs using duration and emotion presented (model with 10 gates). Shown is the estimated percentage of correct recognition of the emotion expressed over time (divided into 10 gates representing the percentage of the full stimulus presented, e.g., 10%, 20%…90%, and 100%). The values were computed with a generalized linear mixed model that evaluated the percentage of correctness using the emotion expressed, the gate duration, and their interaction as predictors. The error bars represent the confidence interval at 95%. The chance level represents the percentage of choosing one of the seven emotions by chance. Continuous thinner lines represent the best polynomial fit based on the orthogonal polynomial contrasts performed in Table 3 (anger: cubic, disgust: quadratic, fear: quadratic, sadness: quadratic, joy: linear, neutral: quadratic). The recognition of the neutral emotion at the early gates revealed that judged neutrality was on average selected by the participants more often than were the other emotions, causing a clear bias (at the first 25% of total duration presented, participants selected the following as the preferred response: neutral: 648, anger: 208, surprise: 279, fear: 386, joy: 134, sadness: 172, disgust: 124). The confusion matrix also showed this bias with a more important proportion of responses for neutral than for the other choices (S2 Table). To control for this bias, we calculated the unbiased rating (Hu Scores) for each gate and each emotion (Fig 1). (PDF) [file pone.0206216.s004.pdf]
