# Supplementary material for: Unfolding and dynamics of affect bursts decoding in humans
Source: PLoS One. 2018 Oct 30;13(10):e0206216. doi: 10.1371/journal.pone.0206216 (PMC6207317; doi:10.1371/journal.pone.0206216)

Estimation of the input value for specific emotions for every 50ms increments. We used separate emotions to estimate the input continuous value (0-100) using linear mixed models.

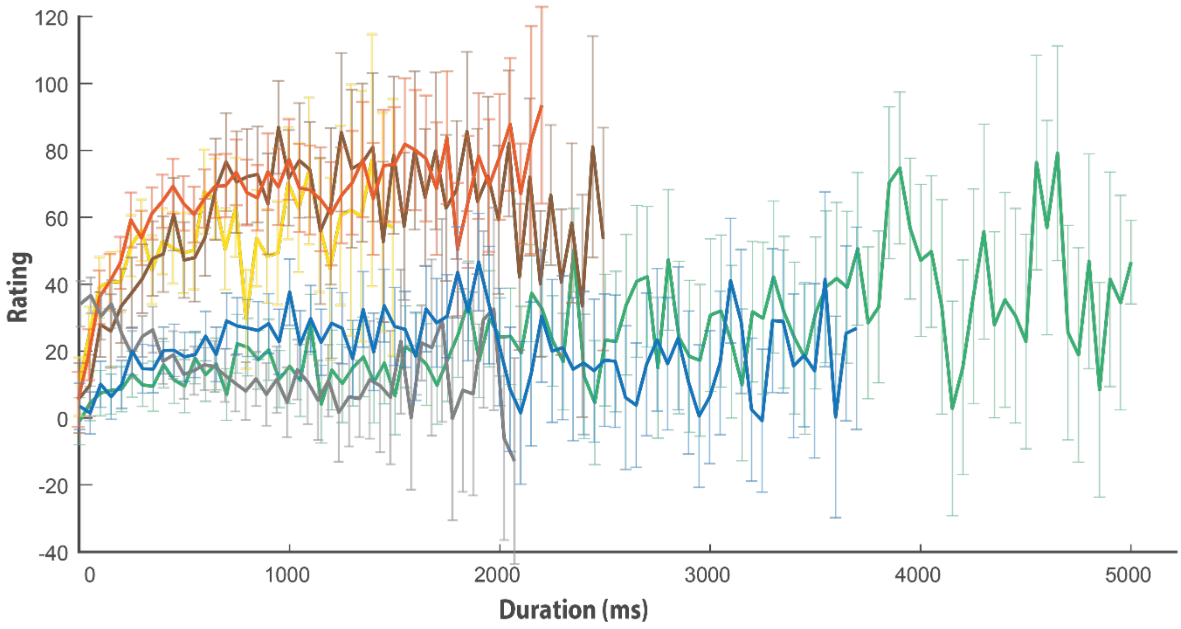

Supplement: S3 Fig — Estimation of the input value for specific emotions for every 50ms increments. We used separate emotions to estimate the input continuous value (0-100) using linear mixed models. (PDF) [file pone.0206216.s005.pdf]
