# Supplementary material for: Unfolding and dynamics of affect bursts decoding in humans
Source: PLoS One. 2018 Oct 30;13(10):e0206216. doi: 10.1371/journal.pone.0206216 (PMC6207317; doi:10.1371/journal.pone.0206216)

Model using only a sub-set of stimuli from different emotions that are matched for recognition at full-length. This was computed in order to rule out the possibility that differences in recognition at earlier time-points merely reflects the ease of recognition of the tokens of different emotions in this stimulus set. Recognition curves reveal a similar pattern with anger, disgust, and fear being recognized more accurately at earlier gates, while sadness and joy are less recognized at earlier gates. Neutral is not depicted here due to its biased nature in our study.

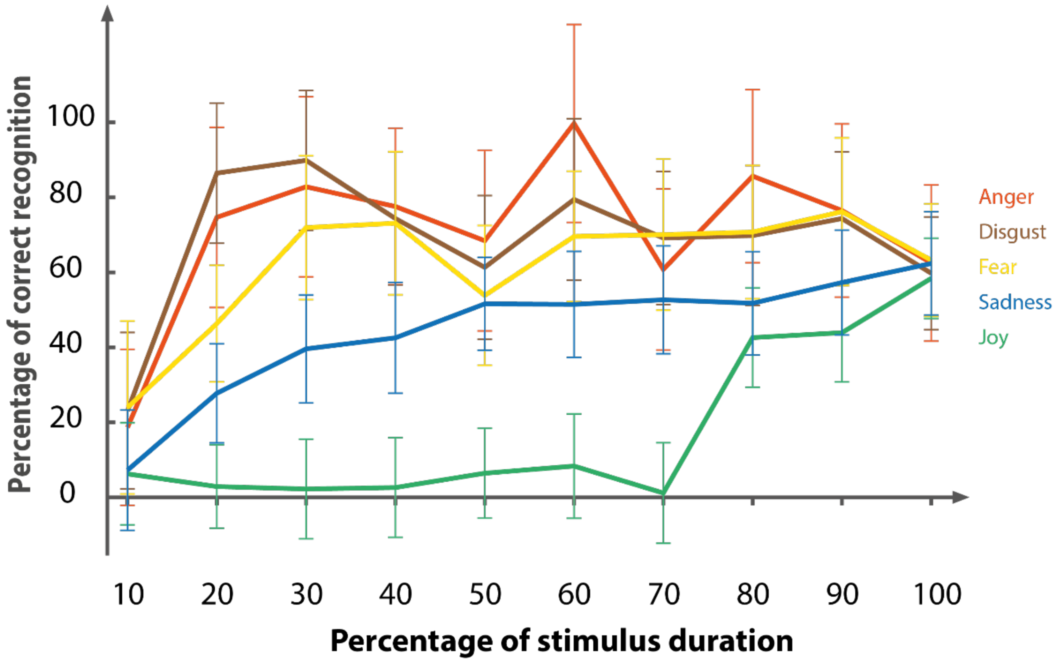

Supplement: S4 Fig — Model using only a sub-set of stimuli from different emotions that are matched for recognition at full-length. This was computed in order to rule out the possibility that differences in recognition at earlier time-points merely reflects the ease of recognition of the tokens of different emotions in this stimulus set. Recognition curves reveal a similar pattern with anger, disgust, and fear being recognized more accurately at earlier gates, while sadness and joy are less recognized at earlier gates. Neutral is not depicted here due to its biased nature in our study. (PDF) [file pone.0206216.s006.pdf]
