# Supplementary material for: Unfolding and dynamics of affect bursts decoding in humans
Source: PLoS One. 2018 Oct 30;13(10):e0206216. doi: 10.1371/journal.pone.0206216 (PMC6207317; doi:10.1371/journal.pone.0206216)
Supplement: S5 Fig — Scree plot for the principal component analysis (PCA) for each emotion separately. (PDF) [file pone.0206216.s007.pdf]

Scree Plot for the Principal Component Analysis (PCA) for Each Emotion Separately

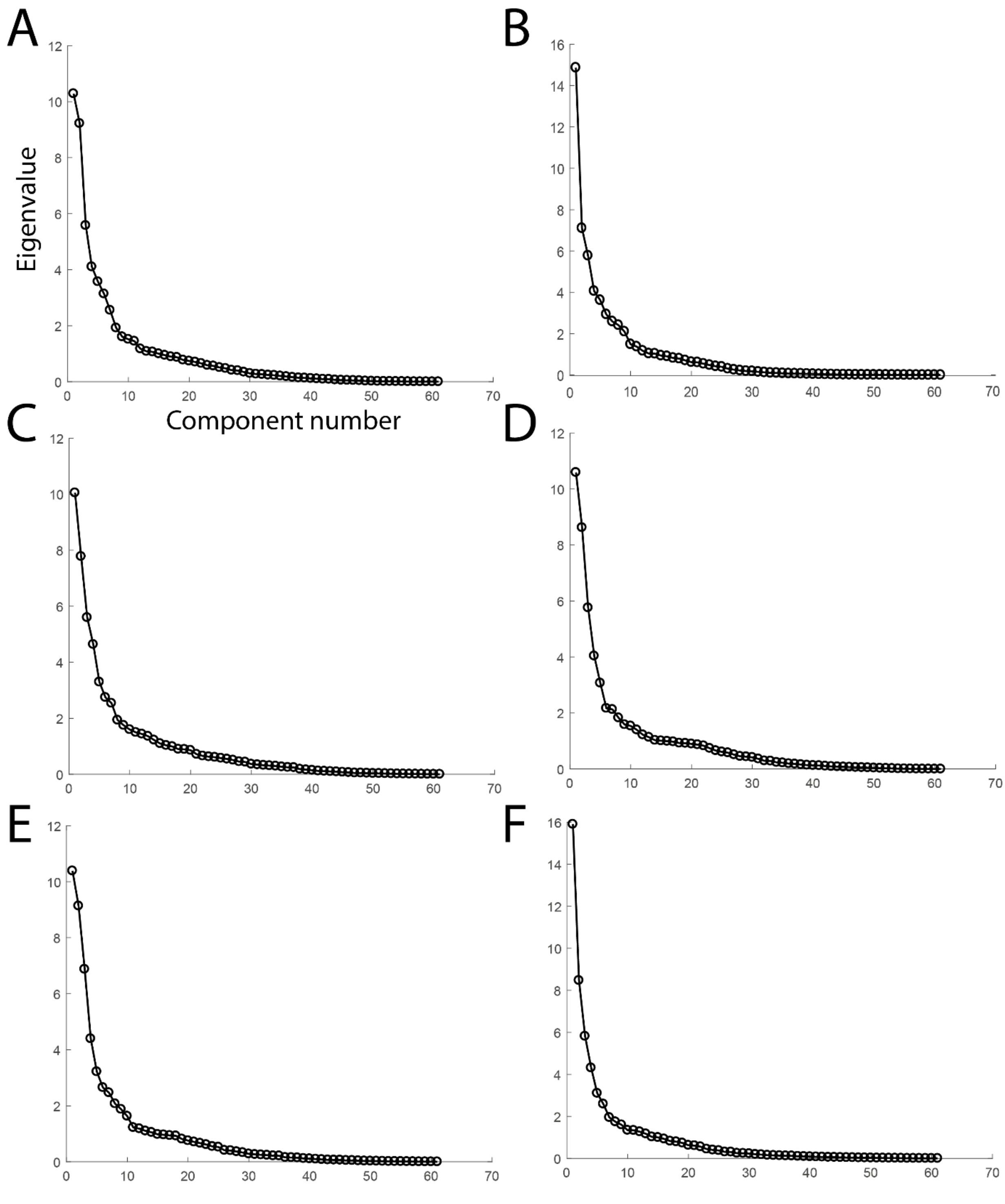

(A) Scree plot for the PCA on stimuli representing anger. (B) Scree plot for the PCA on stimuli representing disgust. (C) Scree plot for the PCA on stimuli representing fear. (D) Scree plot for the PCA on stimuli representing joy. (E) Scree plot for the PCA on stimuli representing neutral. (F) Scree plot for the PCA on stimuli representing sadness.
