# Supplementary material for: Unfolding and dynamics of affect bursts decoding in humans
Source: PLoS One. 2018 Oct 30;13(10):e0206216. doi: 10.1371/journal.pone.0206216 (PMC6207317; doi:10.1371/journal.pone.0206216)
Supplement: S1 Table — Mean and Range of Total Duration and Number of Subdivisions for Each Emotion Separately. (PDF) [file pone.0206216.s008.pdf]

Mean and Range of Total Duration and Number of Subdivisions for Each Emotion Separately

	Total duration	Number of subdivisions
Anger	M = 1070ms [400ms; 2250ms]	M = 21.4 [8; 45]
Disgust	M = 1070ms [350ms; 2550ms]	M = 21.4 [7; 51]
Fear	M = 715ms [200ms; 1550ms]	M = 14.3 [4; 31]
Joy	M = 1512.5ms [350ms; 5050ms]	M = 30.25 [7; 101]
Neutral	M = 1015ms [350ms; 2150ms]	M = 20.3 [7; 43]
Sadness	M = 1277.5ms [200ms; 3750ms]	M = 25.55 [4; 75]
