# Supplementary material for: Unfolding and dynamics of affect bursts decoding in humans
Source: PLoS One. 2018 Oct 30;13(10):e0206216. doi: 10.1371/journal.pone.0206216 (PMC6207317; doi:10.1371/journal.pone.0206216)
Supplement: S2 Table — Confusion matrix between the emotion presented and the emotion chosen by participants at different points in time. (PDF) [file pone.0206216.s009.pdf]

Confusion Matrix Between the Emotion Presented and the Emotion Chosen by Participants at Different Points in Time

Duration	Emotion displayed	Emotion response						
		Anger	Fear	Disgust	Joy	Neutral	Sadness	Surprise
10%	Anger	**1314**	621	185	389	1786	116	802
10%	Fear	160	**1329**	8	266	1046	196	352
10%	Disgust	256	649	**690**	90	774	169	522
10%	Joy	567	4380	531	**655**	2376	402	2849
10%	Neutral	8	28	53	289	**3616**	202	533
10%	Sadness	35	235	96	311	4900	**925**	701
20%	Anger	**5643**	1744	500	102	1210	130	752
20%	Fear	537	**3165**	140	409	628	489	1170
20%	Disgust	154	630	**2407**	2	619	200	560
20%	Joy	735	5596	990	**1641**	1349	1695	2564
20%	Neutral	67	180	62	911	**5453**	314	2421
20%	Sadness	32	581	351	999	5155	**1727**	453
30%	Anger	**7151**	1113	405	136	243	226	349
30%	Fear	600	**3492**	356	257	505	321	1078
30%	Disgust	200	226	**2857**	102	152	252	267
30%	Joy	759	6298	1228	**1290**	540	2168	2834
30%	Neutral	134	102	98	741	**3321**	333	2880
30%	Sadness	24	925	272	718	4685	**2438**	566
40%	Anger	**8142**	1878	611	206	173	276	624
40%	Fear	355	**4058**	246	495	308	243	848
40%	Disgust	251	468	**3611**	207	119	72	424
40%	Joy	1041	5434	1017	**2292**	485	3018	1750
40%	Neutral	2	208	385	1257	**2374**	300	3813
40%	Sadness	29	1531	553	1449	2921	**2882**	482
50%	Anger	**9808**	1557	687	413	99	130	704
50%	Fear	791	**6016**	199	287	373	483	1186
50%	Disgust	189	460	**4591**	10	117	331	417
50%	Joy	2100	4874	1337	**3556**	626	3774	3295
50%	Neutral	3	43	494	2620	**3769**	789	4941
50%	Sadness	90	1138	592	1589	3350	**4731**	592
60%	Anger	**8616**	1827	444	144	132	234	331
60%	Fear	892	**4606**	217	311	176	482	829
60%	Disgust	178	277	**4134**	140	209	165	232
60%	Joy	1678	3570	1299	**2290**	898	3363	1663
60%	Neutral	9	81	390	1592	**2496**	332	3493
60%	Sadness	84	1342	502	1728	2894	**3869**	532
70%	Anger	**8901**	1598	669	231	8	149	578
70%	Fear	816	**4641**	306	250	128	260	542
70%	Disgust	75	98	**4546**	179	74	358	328
70%	Joy	1905	3356	1585	**4123**	692	2793	1358
70%	Neutral	58	102	246	1794	**1883**	511	4119
70%	Sadness	64	1353	658	972	2873	**3636**	1016
80%	Anger	**9746**	1020	527	12	71	198	651
80%	Fear	1412	**5441**	336	230	0	532	1613
80%	Disgust	135	127	**4645**	88	103	254	161

80%	Joy	1066	2902	1795	**4838**	[illegible]	[illegible]	[illegible]
80%	Neutral	25	160	184	2669	**1557**	287	4296
80%	Sadness	103	1290	665	2157	2235	**3526**	986
90%	Anger	**8799**	1038	304	83	88	154	601
90%	Fear	860	**4968**	585	293	127	517	719
90%	Disgust	305	72	**4101**	325	192	204	250
90%	Joy	725	2949	1285	**5700**	325	3714	1896
90%	Neutral	153	24	438	2054	**1965**	495	4161
90%	Sadness	8	994	451	1587	2844	**3933**	503
100%	Anger	**13071**	1420	743	101	81	250	547
100%	Fear	1303	**7733**	496	56	6	1434	1039
100%	Disgust	376	400	**6534**	194	441	413	440
100%	Joy	1452	4038	2108	**8923**	427	4419	2198
100%	Neutral	149	9	607	5031	**2709**	524	6997
100%	Sadness	122	1244	702	2141	3320	**5552**	557
