# Supplementary material for: Unfolding and dynamics of affect bursts decoding in humans
Source: PLoS One. 2018 Oct 30;13(10):e0206216. doi: 10.1371/journal.pone.0206216 (PMC6207317; doi:10.1371/journal.pone.0206216)
Supplement: S3 Table — Loading Factors of the Principal Component Analysis for the Six Different Emotions Taken Individually. (PDF) [file pone.0206216.s010.pdf]

Loading Factors of the Principal Component Analysis for the Six Different Emotions Taken Individually

Anger

| Acoustic Feature | PC 1 (16.8%) | PC 2 (15.1%) | PC 3 (9.1%) | PC 4 (4.1%) |
|---|---|---|---|---|
| alphaRatioUV_sma3nz_amean | 0.60 | **0.46** | 0.06 | -0.29 |
| alphaRatioV_sma3nz_amean | **0.71** | -0.10 | 0.06 | **0.43** |
| alphaRatioV_sma3nz_stddevNorm | 0.08 | 0.12 | 0.14 | 0.19 |
| F0semitoneFrom27.5Hz_sma3nz_amean | **0.90** | -0.08 | -0.34 | 0.11 |
| F0semitoneFrom27.5Hz_sma3nz_meanFallingSlope | -0.24 | -0.23 | 0.38 | **0.41** |
| F0semitoneFrom27.5Hz_sma3nz_meanRisingSlope | -0.09 | **-0.82** | 0.10 | -0.04 |
| F0semitoneFrom27.5Hz_sma3nz_pctlrange0-2 | 0.04 | -0.58 | 0.41 | 0.29 |
| F0semitoneFrom27.5Hz_sma3nz_percentile20.0 | **0.83** | 0.11 | **-0.43** | 0.03 |
| F0semitoneFrom27.5Hz_sma3nz_percentile50.0 | **0.87** | -0.15 | -0.36 | 0.07 |
| F0semitoneFrom27.5Hz_sma3nz_percentile80.0 | **0.87** | -0.26 | -0.19 | 0.21 |
| F0semitoneFrom27.5Hz_sma3nz_stddevFallingSlope | -0.09 | -0.44 | 0.20 | -0.17 |
| F0semitoneFrom27.5Hz_sma3nz_stddevNorm | -0.24 | -0.59 | **0.47** | 0.36 |
| F0semitoneFrom27.5Hz_sma3nz_stddevRisingSlope | 0.15 | -0.26 | 0.41 | 0.07 |
| F1amplitudeLogRelF0_sma3nz_amean | 0.08 | -0.01 | -0.11 | -0.06 |
| F1amplitudeLogRelF0_sma3nz_stddevNorm | 0.09 | 0.03 | 0.02 | -0.02 |
| F1bandwidth_sma3nz_amean | **-0.51** | 0.12 | 0.00 | -0.05 |
| F1bandwidth_sma3nz_stddevNorm | 0.39 | 0.07 | 0.12 | 0.18 |
| F1frequency_sma3nz_amean | 0.51 | -0.30 | -0.23 | **0.44** |
| F1frequency_sma3nz_stddevNorm | 0.44 | -0.16 | 0.08 | **-0.59** |
| F2amplitudeLogRelF0_sma3nz_amean | 0.34 | **0.68** | 0.47 | 0.28 |
| F2amplitudeLogRelF0_sma3nz_stddevNorm | 0.11 | -0.13 | 0.12 | -0.06 |
| F2frequency_sma3nz_amean | 0.67 | -0.32 | -0.26 | **0.39** |
| F2frequency_sma3nz_stddevNorm | 0.28 | -0.32 | -0.07 | **-0.66** |
| F3amplitudeLogRelF0_sma3nz_amean | 0.51 | **0.61** | 0.41 | 0.22 |
| F3amplitudeLogRelF0_sma3nz_stddevNorm | -0.01 | 0.10 | -0.29 | -0.29 |
| F3frequency_sma3nz_amean | 0.64 | -0.28 | -0.22 | 0.35 |
| F3frequency_sma3nz_stddevNorm | 0.38 | -0.47 | -0.08 | **-0.39** |
| hammarbergIndexUV_sma3nz_amean | **-0.59** | -0.62 | -0.10 | 0.22 |
| hammarbergIndexV_sma3nz_amean | **-0.63** | 0.18 | **-0.36** | 0.08 |
| hammarbergIndexV_sma3nz_stddevNorm | 0.36 | -0.35 | 0.40 | **-0.40** |
| HNRdBACF_sma3nz_amean | 0.21 | **0.50** | -0.29 | -0.26 |
| HNRdBACF_sma3nz_stddevNorm | 0.01 | 0.08 | -0.05 | -0.05 |
| jitterLocal_sma3nz_amean | -0.26 | -0.68 | 0.14 | 0.38 |
| jitterLocal_sma3nz_stddevNorm | -0.07 | 0.00 | **0.51** | -0.05 |
| logRelF0-H1-A3_sma3nz_amean | -0.27 | -0.10 | **-0.62** | -0.10 |
| logRelF0-H1-A3_sma3nz_stddevNorm | 0.05 | -0.02 | **0.56** | 0.26 |
| logRelF0-H1-H2_sma3nz_amean | 0.07 | -0.26 | **-0.53** | -0.06 |
| logRelF0-H1-H2_sma3nz_stddevNorm | -0.02 | -0.29 | -0.21 | -0.03 |
| loudness_sma3_amean | 0.62 | -0.41 | 0.40 | -0.17 |
| loudness_sma3_meanFallingSlope | -0.14 | -0.31 | 0.28 | -0.16 |
| loudness_sma3_meanRisingSlope | 0.04 | -0.52 | 0.16 | -0.18 |
| loudness_sma3_pctlrange0-2 | 0.06 | **-0.83** | -0.15 | -0.14 |
| loudness_sma3_percentile20.0 | 0.57 | 0.10 | 0.47 | -0.08 |
| loudness_sma3_percentile50.0 | 0.63 | [illegible] | 0.37 | [illegible] |

| | | | | |
|---|---|---|---|---|
| loudness_sma3_percentile80.0 | 0.50 | -0.71 | 0.23 | -0.20 |
| loudness_sma3_stddevFallingSlope | -0.20 | 0.04 | **0.55** | 0.04 |
| loudness_sma3_stddevNorm | -0.06 | **-0.72** | -0.35 | -0.03 |
| loudness_sma3_stddevRisingSlope | 0.04 | -0.72 | 0.20 | -0.35 |
| loudnessPeaksPerSec | -0.36 | 0.20 | 0.34 | -0.25 |
| MeanUnvoicedSegmentLength | -0.16 | **-0.72** | -0.24 | -0.22 |
| MeanVoicedSegmentLengthSec | 0.06 | 0.14 | **0.50** | -0.28 |
| shimmerLocaldB_sma3nz_amean | -0.15 | -0.23 | 0.19 | 0.10 |
| shimmerLocaldB_sma3nz_stddevNorm | 0.15 | 0.09 | 0.29 | -0.04 |
| slopeUV0-500_sma3nz_amean | **-0.38** | **-0.75** | -0.17 | 0.00 |
| slopeUV500-1500_sma3nz_amean | 0.56 | 0.19 | -0.01 | **-0.40** |
| slopeV0-500_sma3nz_amean | 0.02 | **0.20** | 0.12 | -0.29 |
| slopeV0-500_sma3nz_stddevNorm | 0.19 | -0.03 | -0.22 | 0.19 |
| slopeV500-1500_sma3nz_amean | 0.62 | -0.09 | 0.02 | **0.47** |
| slopeV500-1500_sma3nz_stddevNorm | -0.03 | -0.03 | -0.01 | 0.10 |
| StddevVoicedSegmentLengthSec | **-0.53** | -0.36 | 0.02 | 0.33 |
| VoicedSegmentsPerSec | -0.16 | 0.06 | **-0.38** | 0.19 |

Disgust

| Acoustic Feature | PC 1 (24.3%) | PC 2 (11.6%) | PC 3 (9.4%) | PC 4 (6.6%) |
|---|---|---|---|---|
| alphaRatioUV_sma3nz_amean | **0.83** | -0.10 | -0.34 | -0.15 |
| alphaRatioV_sma3nz_amean | -0.28 | 0.15 | 0.00 | -0.16 |
| alphaRatioV_sma3nz_stddevNorm | -0.14 | -0.01 | **0.40** | 0.14 |
| F0semitoneFrom27.5Hz_sma3nz_amean | 0.60 | -0.51 | -0.17 | 0.22 |
| F0semitoneFrom27.5Hz_sma3nz_meanFallingSlope | -0.72 | -0.23 | -0.10 | 0.22 |
| F0semitoneFrom27.5Hz_sma3nz_meanRisingSlope | -0.47 | **-0.69** | -0.36 | -0.10 |
| F0semitoneFrom27.5Hz_sma3nz_pctlrange0-2 | -0.68 | -0.47 | -0.35 | -0.11 |
| F0semitoneFrom27.5Hz_sma3nz_percentile20.0 | **0.84** | -0.06 | 0.11 | 0.21 |
| F0semitoneFrom27.5Hz_sma3nz_percentile50.0 | 0.61 | -0.39 | -0.19 | 0.22 |
| F0semitoneFrom27.5Hz_sma3nz_percentile80.0 | 0.05 | **-0.71** | -0.35 | 0.10 |
| F0semitoneFrom27.5Hz_sma3nz_stddevFallingSlope | **-0.81** | -0.01 | 0.00 | 0.15 |
| F0semitoneFrom27.5Hz_sma3nz_stddevNorm | -0.76 | -0.38 | -0.41 | -0.10 |
| F0semitoneFrom27.5Hz_sma3nz_stddevRisingSlope | -0.57 | **-0.57** | -0.34 | 0.01 |
| F1amplitudeLogRelF0_sma3nz_amean | 0.00 | -0.05 | -0.10 | 0.13 |
| F1amplitudeLogRelF0_sma3nz_stddevNorm | -0.58 | 0.12 | 0.10 | -0.03 |
| F1bandwidth_sma3nz_amean | -0.36 | **0.72** | -0.23 | -0.16 |
| F1bandwidth_sma3nz_stddevNorm | 0.01 | -0.22 | **-0.59** | 0.28 |
| F1frequency_sma3nz_amean | 0.03 | -0.03 | -0.23 | **-0.51** |
| F1frequency_sma3nz_stddevNorm | -0.14 | -0.21 | **-0.69** | 0.16 |
| F2amplitudeLogRelF0_sma3nz_amean | -0.07 | 0.01 | -0.36 | **-0.72** |
| F2amplitudeLogRelF0_sma3nz_stddevNorm | -0.36 | -0.06 | 0.11 | -0.04 |
| F2frequency_sma3nz_amean | 0.06 | -0.32 | -0.16 | **-0.56** |
| F2frequency_sma3nz_stddevNorm | -0.01 | -0.51 | -0.32 | **0.42** |
| F3amplitudeLogRelF0_sma3nz_amean | 0.05 | -0.10 | -0.46 | **-0.64** |
| F3amplitudeLogRelF0_sma3nz_stddevNorm | 0.49 | 0.07 | **0.29** | -0.10 |
| F3frequency_sma3nz_amean | 0.26 | -0.42 | 0.20 | -0.23 |
| F3frequency_sma3nz_stddevNorm | -0.50 | 0.20 | **-0.66** | 0.03 |
| hammarbergIndexUV_sma3nz_amean | **-0.87** | 0.12 | 0.27 | 0.10 |
| hammarbergIndexV_sma3nz_amean | -0.46 | **0.51** | -0.04 | -0.14 |
| hammarbergIndexV_sma3nz_stddevNorm | -0.10 | -0.45 | -0.23 | **0.58** |

| | | | | |
|---|---|---|---|---|
| HNRdBACF_sma3nz_amean | **0.71** | 0.40 | [illegible] | [illegible] |
| HNRdBACF_sma3nz_stddevNorm | -0.02 | -0.10 | -0.05 | 0.03 |
| jitterLocal_sma3nz_amean | -0.68 | -0.49 | -0.10 | -0.11 |
| jitterLocal_sma3nz_stddevNorm | 0.27 | 0.15 | -0.47 | 0.14 |
| logRelF0-H1-A3_sma3nz_amean | 0.30 | -0.35 | 0.28 | **0.35** |
| logRelF0-H1-A3_sma3nz_stddevNorm | -0.55 | 0.15 | -0.51 | 0.07 |
| logRelF0-H1-H2_sma3nz_amean | -0.16 | **-0.60** | -0.05 | 0.17 |
| logRelF0-H1-H2_sma3nz_stddevNorm | -0.04 | -0.23 | -0.07 | -0.03 |
| loudness_sma3_amean | 0.20 | **0.57** | -0.46 | 0.26 |
| loudness_sma3_meanFallingSlope | -0.27 | 0.08 | -0.15 | 0.10 |
| loudness_sma3_meanRisingSlope | -0.07 | 0.16 | -0.11 | **0.40** |
| loudness_sma3_pctlrange0-2 | **-0.85** | 0.10 | 0.08 | 0.08 |
| loudness_sma3_percentile20.0 | **0.65** | 0.39 | -0.38 | 0.19 |
| loudness_sma3_percentile50.0 | 0.35 | **0.50** | **-0.54** | 0.14 |
| loudness_sma3_percentile80.0 | -0.40 | **0.51** | -0.29 | 0.28 |
| loudness_sma3_stddevFallingSlope | -0.41 | 0.06 | -0.14 | 0.35 |
| loudness_sma3_stddevNorm | **-0.91** | 0.02 | 0.07 | 0.05 |
| loudness_sma3_stddevRisingSlope | -0.24 | 0.15 | -0.10 | **0.55** |
| loudnessPeaksPerSec | 0.49 | -0.05 | 0.17 | 0.27 |
| MeanUnvoicedSegmentLength | **-0.84** | 0.14 | 0.17 | 0.04 |
| MeanVoicedSegmentLengthSec | -0.23 | 0.25 | **-0.54** | -0.10 |
| shimmerLocaldB_sma3nz_amean | -0.63 | -0.45 | **0.30** | -0.15 |
| shimmerLocaldB_sma3nz_stddevNorm | 0.00 | 0.42 | -0.38 | -0.02 |
| slopeUV0-500_sma3nz_amean | -0.75 | 0.08 | **0.38** | 0.18 |
| slopeUV500-1500_sma3nz_amean | **0.83** | -0.11 | -0.34 | -0.14 |
| slopeV0-500_sma3nz_amean | 0.42 | **-0.57** | -0.09 | -0.02 |
| slopeV0-500_sma3nz_stddevNorm | -0.37 | 0.28 | 0.00 | 0.35 |
| slopeV500-1500_sma3nz_amean | -0.20 | 0.47 | -0.19 | **-0.37** |
| slopeV500-1500_sma3nz_stddevNorm | 0.06 | -0.15 | 0.19 | -0.08 |
| StddevVoicedSegmentLengthSec | -0.76 | 0.14 | 0.17 | 0.02 |
| VoicedSegmentsPerSec | 0.24 | -0.13 | **0.52** | 0.05 |

## Fear

| Acoustic Feature | PC 1 (16.2%) | PC 2 (12.5%) | PC 3 (9.1%) | PC 4 (7.5%) |
|---|---|---|---|---|
| alphaRatioUV_sma3nz_amean | 0.28 | **-0.74** | -0.36 | 0.12 |
| alphaRatioV_sma3nz_amean | -0.34 | 0.02 | -0.48 | -0.10 |
| alphaRatioV_sma3nz_stddevNorm | 0.04 | -0.02 | 0.00 | -0.04 |
| F0semitoneFrom27.5Hz_sma3nz_amean | -0.31 | **0.52** | **-0.70** | -0.23 |
| F0semitoneFrom27.5Hz_sma3nz_meanFallingSlope | -0.36 | -0.21 | 0.08 | 0.09 |
| F0semitoneFrom27.5Hz_sma3nz_meanRisingSlope | -0.16 | -0.18 | -0.16 | -0.30 |
| F0semitoneFrom27.5Hz_sma3nz_pctlrange0-2 | -0.50 | -0.42 | 0.29 | 0.00 |
| F0semitoneFrom27.5Hz_sma3nz_percentile20.0 | -0.07 | **0.60** | **-0.70** | -0.24 |
| F0semitoneFrom27.5Hz_sma3nz_percentile50.0 | -0.40 | 0.43 | **-0.65** | -0.27 |
| F0semitoneFrom27.5Hz_sma3nz_percentile80.0 | -0.46 | 0.38 | **-0.60** | -0.27 |
| F0semitoneFrom27.5Hz_sma3nz_stddevFallingSlope | -0.37 | -0.23 | 0.08 | -0.08 |
| F0semitoneFrom27.5Hz_sma3nz_stddevNorm | -0.40 | -0.56 | 0.30 | -0.31 |
| F0semitoneFrom27.5Hz_sma3nz_stddevRisingSlope | -0.26 | -0.15 | -0.11 | -0.17 |
| F1amplitudeLogRelF0_sma3nz_amean | -0.06 | -0.01 | -0.17 | -0.12 |
| F1amplitudeLogRelF0_sma3nz_stddevNorm | 0.17 | -0.18 | -0.09 | -0.08 |
| F1bandwidth_sma3nz_amean | **0.68** | -0.05 | 0.14 | **0.27** |

| | | | | |
|---|---|---|---|---|
| F1bandwidth_sma3nz_stddevNorm | -0.60 | [illegible] | [illegible] | 0.19 |
| F1frequency_sma3nz_amean | 0.17 | 0.13 | 0.22 | **-0.83** |
| F1frequency_sma3nz_stddevNorm | -0.26 | -0.18 | -0.39 | **0.60** |
| F2amplitudeLogRelF0_sma3nz_amean | 0.01 | -0.04 | -0.14 | -0.14 |
| F2amplitudeLogRelF0_sma3nz_stddevNorm | 0.21 | 0.13 | -0.04 | 0.02 |
| F2frequency_sma3nz_amean | -0.10 | 0.18 | 0.00 | **-0.71** |
| F2frequency_sma3nz_stddevNorm | -0.32 | -0.11 | -0.30 | **0.36** |
| F3amplitudeLogRelF0_sma3nz_amean | 0.22 | **-0.79** | -0.07 | 0.03 |
| F3amplitudeLogRelF0_sma3nz_stddevNorm | 0.07 | -0.03 | 0.02 | -0.14 |
| F3frequency_sma3nz_amean | 0.17 | 0.07 | 0.20 | **-0.85** |
| F3frequency_sma3nz_stddevNorm | -0.16 | -0.10 | -0.49 | **0.32** |
| hammarbergIndexUV_sma3nz_amean | -0.40 | **0.74** | **0.43** | -0.10 |
| hammarbergIndexV_sma3nz_amean | **0.72** | 0.20 | 0.23 | -0.11 |
| hammarbergIndexV_sma3nz_stddevNorm | **-0.79** | -0.27 | 0.16 | 0.12 |
| HNRdBACF_sma3nz_amean | 0.28 | 0.21 | -0.44 | -0.42 |
| HNRdBACF_sma3nz_stddevNorm | -0.41 | -0.12 | 0.24 | -0.03 |
| jitterLocal_sma3nz_amean | -0.29 | -0.35 | **0.35** | 0.21 |
| jitterLocal_sma3nz_stddevNorm | -0.37 | -0.41 | -0.04 | **-0.47** |
| logRelF0-H1-A3_sma3nz_amean | 0.19 | 0.39 | -0.52 | -0.03 |
| logRelF0-H1-A3_sma3nz_stddevNorm | -0.37 | -0.36 | 0.30 | -0.16 |
| logRelF0-H1-H2_sma3nz_amean | -0.22 | 0.13 | **-0.52** | 0.20 |
| logRelF0-H1-H2_sma3nz_stddevNorm | -0.16 | 0.19 | 0.09 | 0.19 |
| loudness_sma3_amean | **-0.69** | -0.41 | -0.01 | -0.14 |
| loudness_sma3_meanFallingSlope | -0.41 | 0.04 | 0.14 | 0.01 |
| loudness_sma3_meanRisingSlope | -0.44 | 0.05 | -0.11 | 0.25 |
| loudness_sma3_pctlrange0-2 | -0.66 | 0.46 | 0.07 | 0.25 |
| loudness_sma3_percentile20.0 | -0.13 | **-0.73** | -0.06 | -0.32 |
| loudness_sma3_percentile50.0 | **-0.67** | -0.52 | -0.07 | -0.04 |
| loudness_sma3_percentile80.0 | **-0.82** | -0.04 | 0.03 | 0.03 |
| loudness_sma3_stddevFallingSlope | **-0.71** | -0.07 | 0.11 | 0.13 |
| loudness_sma3_stddevNorm | -0.51 | **0.64** | 0.09 | **0.31** |
| loudness_sma3_stddevRisingSlope | -0.43 | -0.18 | 0.08 | 0.04 |
| loudnessPeaksPerSec | 0.10 | -0.20 | 0.04 | 0.05 |
| MeanUnvoicedSegmentLength | -0.42 | **0.69** | **0.44** | -0.07 |
| MeanVoicedSegmentLengthSec | -0.40 | -0.23 | 0.03 | **-0.52** |
| shimmerLocaldB_sma3nz_amean | 0.37 | 0.26 | **0.31** | 0.22 |
| shimmerLocaldB_sma3nz_stddevNorm | -0.49 | -0.31 | -0.19 | -0.21 |
| slopeUV0-500_sma3nz_amean | **0.49** | **-0.61** | -0.47 | 0.03 |
| slopeUV500-1500_sma3nz_amean | **0.40** | **-0.60** | -0.38 | 0.08 |
| slopeV0-500_sma3nz_amean | **0.77** | -0.01 | 0.13 | -0.06 |
| slopeV0-500_sma3nz_stddevNorm | 0.28 | 0.27 | -0.14 | -0.09 |
| slopeV500-1500_sma3nz_amean | -0.02 | -0.26 | -0.30 | -0.22 |
| slopeV500-1500_sma3nz_stddevNorm | 0.00 | 0.01 | 0.06 | -0.05 |
| StddevUnvoicedSegmentLength | -0.16 | 0.38 | 0.21 | -0.03 |
| StddevVoicedSegmentLengthSec | -0.41 | 0.30 | **0.37** | 0.06 |
| VoicedSegmentsPerSec | 0.20 | 0.11 | -0.26 | 0.23 |

Joy

| Acoustic Feature | PC 1 (17.3%) | PC 2 (14.1%) | PC 3 (9.4%) | PC 4 (6.6%) |
|---|---|---|---|---|
| alphaRatioUV_sma3nz_amean | -0.11 | -0.06 | -0.31 | 0.01 |

| | | | | |
|---|---|---|---|---|
| alphaRatioV_sma3nz_amean | -0.32 | 0.04 | -0.39 | -0.20 |
| alphaRatioV_sma3nz_stddevNorm | 0.03 | 0.05 | 0.05 | 0.04 |
| F0semitoneFrom27.5Hz_sma3nz_amean | **-0.88** | **0.31** | 0.06 | 0.13 |
| F0semitoneFrom27.5Hz_sma3nz_meanFallingSlope | -0.40 | **-0.69** | -0.24 | -0.19 |
| F0semitoneFrom27.5Hz_sma3nz_meanRisingSlope | -0.31 | -0.54 | 0.05 | -0.05 |
| F0semitoneFrom27.5Hz_sma3nz_pctlrange0-2 | 0.14 | -0.52 | 0.25 | **0.31** |
| F0semitoneFrom27.5Hz_sma3nz_percentile20.0 | **-0.85** | **0.39** | 0.00 | 0.05 |
| F0semitoneFrom27.5Hz_sma3nz_percentile50.0 | **-0.87** | **0.31** | 0.07 | 0.15 |
| F0semitoneFrom27.5Hz_sma3nz_percentile80.0 | **-0.87** | 0.19 | 0.11 | 0.20 |
| F0semitoneFrom27.5Hz_sma3nz_stddevFallingSlope | -0.36 | **-0.69** | -0.24 | -0.25 |
| F0semitoneFrom27.5Hz_sma3nz_stddevNorm | 0.21 | **-0.69** | 0.22 | 0.28 |
| F0semitoneFrom27.5Hz_sma3nz_stddevRisingSlope | -0.29 | -0.49 | 0.02 | 0.12 |
| F1amplitudeLogRelF0_sma3nz_amean | 0.10 | 0.07 | -0.20 | 0.06 |
| F1amplitudeLogRelF0_sma3nz_stddevNorm | -0.16 | 0.01 | -0.11 | -0.11 |
| F1bandwidth_sma3nz_amean | **0.80** | -0.23 | -0.19 | -0.09 |
| F1bandwidth_sma3nz_stddevNorm | -0.33 | -0.37 | -0.03 | **0.49** |
| F1frequency_sma3nz_amean | -0.74 | 0.28 | 0.11 | -0.26 |
| F1frequency_sma3nz_stddevNorm | -0.40 | -0.38 | -0.17 | 0.30 |
| F2amplitudeLogRelF0_sma3nz_amean | -0.11 | 0.02 | -0.01 | 0.22 |
| F2amplitudeLogRelF0_sma3nz_stddevNorm | 0.03 | -0.12 | -0.10 | -0.05 |
| F2frequency_sma3nz_amean | **-0.76** | 0.25 | 0.21 | -0.20 |
| F2frequency_sma3nz_stddevNorm | -0.68 | -0.36 | 0.03 | 0.05 |
| F3amplitudeLogRelF0_sma3nz_amean | -0.09 | 0.10 | -0.51 | 0.12 |
| F3amplitudeLogRelF0_sma3nz_stddevNorm | -0.12 | 0.06 | -0.02 | 0.20 |
| F3frequency_sma3nz_amean | -0.75 | 0.16 | -0.03 | -0.31 |
| F3frequency_sma3nz_stddevNorm | 0.12 | -0.49 | -0.21 | 0.30 |
| hammarbergIndexUV_sma3nz_amean | -0.43 | -0.56 | -0.18 | -0.33 |
| hammarbergIndexV_sma3nz_amean | **0.55** | -0.18 | **0.38** | -0.09 |
| hammarbergIndexV_sma3nz_stddevNorm | -0.26 | -0.33 | -0.04 | **0.51** |
| HNRdBACF_sma3nz_amean | -0.48 | **0.43** | -0.38 | **0.46** |
| HNRdBACF_sma3nz_stddevNorm | 0.15 | -0.39 | 0.23 | -0.17 |
| jitterLocal_sma3nz_amean | -0.20 | -0.44 | **0.55** | -0.24 |
| jitterLocal_sma3nz_stddevNorm | -0.41 | -0.24 | -0.15 | 0.25 |
| logRelF0-H1-A3_sma3nz_amean | -0.10 | -0.02 | 0.02 | **-0.50** |
| logRelF0-H1-A3_sma3nz_stddevNorm | 0.05 | -0.04 | -0.01 | -0.10 |
| logRelF0-H1-H2_sma3nz_amean | -0.69 | -0.03 | 0.34 | 0.18 |
| logRelF0-H1-H2_sma3nz_stddevNorm | -0.09 | -0.11 | -0.10 | -0.08 |
| loudness_sma3_amean | **0.44** | 0.14 | **-0.74** | 0.00 |
| loudness_sma3_meanFallingSlope | -0.18 | -0.47 | -0.31 | -0.21 |
| loudness_sma3_meanRisingSlope | -0.31 | -0.61 | -0.19 | -0.30 |
| loudness_sma3_pctlrange0-2 | **0.41** | **-0.69** | 0.02 | 0.12 |
| loudness_sma3_percentile20.0 | 0.27 | **0.48** | **-0.67** | -0.02 |
| loudness_sma3_percentile50.0 | 0.24 | 0.20 | **-0.80** | -0.07 |
| loudness_sma3_percentile80.0 | **0.58** | -0.13 | **-0.59** | 0.08 |
| loudness_sma3_stddevFallingSlope | -0.28 | -0.67 | -0.18 | -0.12 |
| loudness_sma3_stddevNorm | 0.15 | **-0.75** | **0.40** | 0.15 |
| loudness_sma3_stddevRisingSlope | -0.34 | -0.61 | -0.35 | -0.10 |
| loudnessPeaksPerSec | -0.19 | 0.17 | 0.20 | **-0.43** |
| MeanUnvoicedSegmentLength | -0.29 | -0.12 | **0.46** | -0.03 |
| MeanVoicedSegmentLengthSec | 0.15 | -0.57 | -0.35 | 0.29 |
| shimmerLocaldB_sma3nz_amean | 0.25 | -0.37 | **0.46** | **-0.41** |

| | | | | |
|---|---|---|---|---|
| shimmerLocaldB_sma3nz_stddevNorm | -0.43 | -0.04 | -0.21 | **0.63** |
| slopeUV0-500_sma3nz_amean | -0.43 | -0.59 | -0.33 | **-0.37** |
| slopeUV500-1500_sma3nz_amean | -0.30 | -0.45 | **-0.57** | -0.29 |
| slopeV0-500_sma3nz_amean | -0.04 | 0.25 | -0.30 | **-0.62** |
| slopeV0-500_sma3nz_stddevNorm | 0.09 | 0.00 | -0.01 | -0.14 |
| slopeV500-1500_sma3nz_amean | -0.62 | 0.28 | -0.40 | -0.16 |
| slopeV500-1500_sma3nz_stddevNorm | -0.12 | 0.02 | -0.10 | -0.04 |
| StddevVoicedSegmentLengthSec | -0.17 | -0.16 | 0.25 | 0.04 |
| VoicedSegmentsPerSec | 0.04 | 0.25 | 0.34 | -0.32 |

## Neutral

| Acoustic Feature | PC 1 (10.4%) | PC 2 (9.1%) | PC 3 (6.8%) | PC 4 (4.4%) |
|---|---|---|---|---|
| alphaRatioUV_sma3nz_amean | 0.43 | 0.20 | **-0.67** | -0.16 |
| alphaRatioV_sma3nz_amean | **-0.77** | 0.25 | **-0.41** | 0.09 |
| alphaRatioV_sma3nz_stddevNorm | 0.40 | **-0.58** | -0.09 | 0.15 |
| F0semitoneFrom27.5Hz_sma3nz_amean | 0.40 | **0.86** | -0.08 | 0.04 |
| F0semitoneFrom27.5Hz_sma3nz_meanFallingSlope | **-0.59** | -0.08 | 0.52 | 0.26 |
| F0semitoneFrom27.5Hz_sma3nz_meanRisingSlope | 0.12 | 0.00 | 0.30 | -0.16 |
| F0semitoneFrom27.5Hz_sma3nz_pctlrange0-2 | -0.08 | 0.10 | 0.57 | -0.05 |
| F0semitoneFrom27.5Hz_sma3nz_percentile20.0 | 0.42 | **0.81** | -0.21 | 0.04 |
| F0semitoneFrom27.5Hz_sma3nz_percentile50.0 | 0.42 | **0.84** | -0.07 | 0.02 |
| F0semitoneFrom27.5Hz_sma3nz_percentile80.0 | 0.39 | **0.86** | 0.04 | 0.02 |
| F0semitoneFrom27.5Hz_sma3nz_stddevFallingSlope | **-0.57** | -0.08 | **0.65** | 0.20 |
| F0semitoneFrom27.5Hz_sma3nz_stddevNorm | -0.17 | -0.11 | **0.73** | 0.03 |
| F0semitoneFrom27.5Hz_sma3nz_stddevRisingSlope | -0.10 | -0.01 | 0.40 | -0.19 |
| F1amplitudeLogRelF0_sma3nz_amean | 0.10 | 0.02 | 0.01 | 0.09 |
| F1amplitudeLogRelF0_sma3nz_stddevNorm | -0.02 | 0.03 | 0.00 | 0.07 |
| F1bandwidth_sma3nz_amean | 0.27 | 0.26 | 0.45 | **0.60** |
| F1bandwidth_sma3nz_stddevNorm | -0.24 | 0.46 | 0.29 | 0.06 |
| F1frequency_sma3nz_amean | **0.71** | 0.49 | 0.18 | -0.13 |
| F1frequency_sma3nz_stddevNorm | -0.07 | 0.42 | 0.32 | -0.11 |
| F2amplitudeLogRelF0_sma3nz_amean | 0.53 | 0.26 | 0.13 | **0.66** |
| F2amplitudeLogRelF0_sma3nz_stddevNorm | 0.06 | -0.01 | -0.01 | -0.01 |
| F2frequency_sma3nz_amean | 0.64 | 0.48 | 0.37 | 0.14 |
| F2frequency_sma3nz_stddevNorm | 0.04 | 0.31 | 0.22 | -0.08 |
| F3amplitudeLogRelF0_sma3nz_amean | 0.54 | 0.28 | 0.12 | **0.66** |
| F3amplitudeLogRelF0_sma3nz_stddevNorm | 0.62 | **-0.58** | -0.28 | 0.01 |
| F3frequency_sma3nz_amean | **0.68** | 0.45 | 0.40 | 0.03 |
| F3frequency_sma3nz_stddevNorm | -0.02 | 0.44 | 0.19 | 0.05 |
| hammarbergIndexUV_sma3nz_amean | **-0.80** | 0.11 | 0.43 | 0.05 |
| hammarbergIndexV_sma3nz_amean | **0.76** | 0.00 | **0.58** | 0.16 |
| hammarbergIndexV_sma3nz_stddevNorm | -0.29 | **0.70** | 0.15 | **-0.24** |
| HNRdBACF_sma3nz_amean | **0.66** | 0.44 | -0.14 | -0.24 |
| HNRdBACF_sma3nz_stddevNorm | 0.11 | 0.03 | -0.15 | 0.00 |
| jitterLocal_sma3nz_amean | -0.15 | -0.02 | 0.46 | 0.06 |
| jitterLocal_sma3nz_stddevNorm | 0.35 | 0.30 | 0.19 | -0.11 |
| logRelF0-H1-A3_sma3nz_amean | **0.65** | **-0.37** | 0.45 | 0.13 |
| logRelF0-H1-A3_sma3nz_stddevNorm | -0.16 | 0.45 | 0.02 | -0.03 |
| logRelF0-H1-H2_sma3nz_amean | 0.49 | **-0.52** | 0.36 | 0.08 |
| logRelF0-H1-H2_sma3nz_stddevNorm | -0.02 | 0.00 | -0.02 | 0.08 |

| | | | | |
|---|---|---|---|---|
| loudness_sma3_amean | [illegible] | [illegible] | [illegible] | [illegible] |
| loudness_sma3_meanFallingSlope | -0.32 | 0.41 | 0.20 | -0.01 |
| loudness_sma3_meanRisingSlope | -0.47 | 0.15 | -0.01 | 0.21 |
| loudness_sma3_pctlrange0-2 | -0.36 | 0.38 | -0.18 | -0.11 |
| loudness_sma3_percentile20.0 | -0.44 | 0.21 | -0.31 | **0.62** |
| loudness_sma3_percentile50.0 | -0.53 | 0.29 | **-0.40** | **0.55** |
| loudness_sma3_percentile80.0 | -0.57 | 0.41 | -0.35 | 0.40 |
| loudness_sma3_stddevFallingSlope | -0.33 | 0.47 | 0.26 | 0.07 |
| loudness_sma3_stddevNorm | -0.17 | 0.34 | -0.11 | **-0.40** |
| loudness_sma3_stddevRisingSlope | -0.12 | 0.46 | 0.18 | 0.04 |
| loudnessPeaksPerSec | -0.26 | -0.28 | 0.13 | 0.51 |
| MeanUnvoicedSegmentLength | **-0.58** | 0.22 | 0.19 | **-0.44** |
| MeanVoicedSegmentLengthSec | 0.19 | 0.44 | 0.13 | 0.01 |
| shimmerLocaldB_sma3nz_amean | -0.15 | 0.04 | -0.10 | 0.46 |
| shimmerLocaldB_sma3nz_stddevNorm | -0.02 | 0.47 | 0.40 | 0.10 |
| slopeUV0-500_sma3nz_amean | 0.57 | **-0.29** | -0.29 | 0.39 |
| slopeUV500-1500_sma3nz_amean | -0.10 | 0.42 | **-0.46** | **-0.39** |
| slopeV0-500_sma3nz_amean | -0.14 | 0.46 | **-0.50** | 0.12 |
| slopeV0-500_sma3nz_stddevNorm | -0.11 | -0.10 | 0.00 | 0.28 |
| slopeV500-1500_sma3nz_amean | -0.26 | 0.31 | 0.01 | **-0.32** |
| slopeV500-1500_sma3nz_stddevNorm | 0.11 | 0.03 | -0.01 | -0.11 |
| StddevUnvoicedSegmentLength | -0.36 | -0.09 | **0.58** | -0.07 |
| StddevVoicedSegmentLengthSec | -0.49 | -0.11 | **0.66** | -0.06 |
| VoicedSegmentsPerSec | 0.08 | -0.28 | -0.19 | 0.33 |

## Sadness

| Acoustic Feature | PC 1 (25.6%) | PC 2 (13.6%) | PC 3 (9.3%) | PC 4 (6.9%) |
|---|---|---|---|---|
| alphaRatioUV_sma3nz_amean | **0.79** | 0.05 | -0.37 | -0.27 |
| alphaRatioV_sma3nz_amean | 0.45 | 0.10 | **0.73** | -0.16 |
| alphaRatioV_sma3nz_stddevNorm | -0.13 | **0.29** | -0.21 | 0.18 |
| F0semitoneFrom27.5Hz_sma3nz_amean | 0.72 | -0.60 | 0.13 | 0.17 |
| F0semitoneFrom27.5Hz_sma3nz_meanFallingSlope | **-0.81** | -0.21 | 0.11 | 0.04 |
| F0semitoneFrom27.5Hz_sma3nz_meanRisingSlope | -0.55 | -0.32 | 0.04 | 0.05 |
| F0semitoneFrom27.5Hz_sma3nz_pctlrange0-2 | -0.52 | -0.29 | 0.06 | 0.38 |
| F0semitoneFrom27.5Hz_sma3nz_percentile20.0 | **0.78** | -0.51 | 0.11 | 0.07 |
| F0semitoneFrom27.5Hz_sma3nz_percentile50.0 | 0.69 | -0.63 | 0.14 | 0.19 |
| F0semitoneFrom27.5Hz_sma3nz_percentile80.0 | 0.64 | **-0.66** | 0.14 | 0.23 |
| F0semitoneFrom27.5Hz_sma3nz_stddevFallingSlope | -0.76 | -0.20 | 0.10 | -0.17 |
| F0semitoneFrom27.5Hz_sma3nz_stddevNorm | **-0.83** | -0.22 | 0.01 | 0.10 |
| F0semitoneFrom27.5Hz_sma3nz_stddevRisingSlope | -0.70 | -0.44 | 0.01 | -0.03 |
| F1amplitudeLogRelF0_sma3nz_amean | -0.05 | 0.00 | 0.02 | -0.04 |
| F1amplitudeLogRelF0_sma3nz_stddevNorm | 0.37 | **0.48** | -0.39 | -0.09 |
| F1bandwidth_sma3nz_amean | 0.20 | -0.50 | **-0.59** | -0.01 |
| F1bandwidth_sma3nz_stddevNorm | -0.23 | **-0.65** | 0.03 | -0.23 |
| F1frequency_sma3nz_amean | 0.35 | **-0.69** | -0.14 | **0.43** |
| F1frequency_sma3nz_stddevNorm | -0.56 | -0.37 | -0.14 | **-0.44** |
| F2amplitudeLogRelF0_sma3nz_amean | 0.51 | -0.54 | -0.44 | -0.23 |
| F2amplitudeLogRelF0_sma3nz_stddevNorm | 0.56 | **0.38** | -0.41 | 0.15 |
| F2frequency_sma3nz_amean | 0.12 | **-0.81** | -0.36 | 0.22 |
| F2frequency_sma3nz_stddevNorm | -0.70 | -0.29 | -0.17 | -0.34 |

| | | | | |
|---|---|---|---|---|
| F3amplitudeLogRelF0_sma3nz_amean | 0.42 | -0.37 | **-0.44** | -0.27 |
| F3amplitudeLogRelF0_sma3nz_stddevNorm | 0.75 | **0.29** | -0.32 | 0.10 |
| F3frequency_sma3nz_amean | 0.09 | **-0.77** | -0.41 | 0.21 |
| F3frequency_sma3nz_stddevNorm | -0.40 | -0.50 | 0.00 | **-0.44** |
| hammarbergIndexUV_sma3nz_amean | **-0.81** | -0.13 | 0.39 | 0.23 |
| hammarbergIndexV_sma3nz_amean | 0.03 | -0.37 | **-0.78** | 0.33 |
| hammarbergIndexV_sma3nz_stddevNorm | -0.23 | -0.51 | 0.25 | -0.34 |
| HNRdBACF_sma3nz_amean | **0.85** | -0.34 | 0.07 | 0.10 |
| HNRdBACF_sma3nz_stddevNorm | 0.20 | 0.05 | 0.34 | -0.26 |
| jitterLocal_sma3nz_amean | **-0.77** | -0.06 | -0.14 | 0.11 |
| jitterLocal_sma3nz_stddevNorm | -0.38 | -0.40 | 0.05 | **-0.45** |
| logRelF0-H1-A3_sma3nz_amean | 0.11 | -0.25 | **-0.66** | 0.06 |
| logRelF0-H1-A3_sma3nz_stddevNorm | -0.05 | -0.20 | -0.04 | -0.25 |
| logRelF0-H1-H2_sma3nz_amean | -0.27 | -0.04 | -0.23 | **-0.61** |
| logRelF0-H1-H2_sma3nz_stddevNorm | 0.02 | -0.10 | 0.02 | 0.05 |
| loudness_sma3_amean | 0.68 | -0.18 | **0.43** | -0.37 |
| loudness_sma3_meanFallingSlope | -0.27 | -0.33 | 0.22 | 0.33 |
| loudness_sma3_meanRisingSlope | 0.09 | -0.22 | **0.53** | **0.44** |
| loudness_sma3_pctlrange0-2 | 0.03 | -0.30 | 0.19 | -0.42 |
| loudness_sma3_percentile20.0 | 0.68 | -0.06 | 0.35 | -0.24 |
| loudness_sma3_percentile50.0 | 0.70 | -0.10 | **0.40** | -0.35 |
| loudness_sma3_percentile80.0 | 0.61 | -0.23 | **0.42** | **-0.46** |
| loudness_sma3_stddevFallingSlope | -0.20 | -0.45 | 0.39 | **0.38** |
| loudness_sma3_stddevNorm | -0.36 | -0.37 | 0.06 | -0.17 |
| loudness_sma3_stddevRisingSlope | -0.11 | -0.48 | 0.31 | **0.38** |
| loudnessPeaksPerSec | 0.03 | 0.24 | 0.29 | **0.42** |
| MeanUnvoicedSegmentLength | **-0.79** | -0.10 | -0.08 | 0.04 |
| MeanVoicedSegmentLengthSec | -0.20 | -0.47 | -0.21 | -0.11 |
| shimmerLocaldB_sma3nz_amean | -0.65 | 0.05 | -0.06 | 0.22 |
| shimmerLocaldB_sma3nz_stddevNorm | -0.01 | -0.26 | 0.13 | -0.27 |
| slopeUV0-500_sma3nz_amean | **0.76** | -0.23 | 0.11 | 0.08 |
| slopeUV500-1500_sma3nz_amean | 0.46 | 0.23 | **-0.59** | -0.25 |
| slopeV0-500_sma3nz_amean | **0.79** | -0.27 | 0.28 | 0.24 |
| slopeV0-500_sma3nz_stddevNorm | 0.23 | -0.14 | -0.04 | 0.20 |
| slopeV500-1500_sma3nz_amean | -0.03 | -0.09 | 0.35 | -0.01 |
| slopeV500-1500_sma3nz_stddevNorm | 0.00 | 0.06 | -0.01 | 0.02 |
| StddevUnvoicedSegmentLength | -0.26 | -0.09 | 0.17 | -0.15 |
| StddevVoicedSegmentLengthSec | -0.64 | -0.24 | -0.06 | 0.02 |
| VoicedSegmentsPerSec | 0.20 | **0.31** | -0.10 | 0.18 |

*Note.* The features highlighted represent the most salient acoustic features included in the best PC (up to five of the most salient acoustic features for both positive and negative weights). The parentheses next to the PC show the percentage of variance explained by the PC. See scree plot in Appendix I.
