# Supplementary material for: Unfolding and dynamics of affect bursts decoding in humans
Source: PLoS One. 2018 Oct 30;13(10):e0206216. doi: 10.1371/journal.pone.0206216 (PMC6207317; doi:10.1371/journal.pone.0206216)
Supplement: S4 Table — Contrast computations between a specific scale when the corresponding emotion is presented and all other scales. (PDF) [file pone.0206216.s011.pdf]

Contrast Computations Between a Specific Scale When the Corresponding Emotion Is Presented and All Other Scales

	Chi-squared test
Anger	χ^2 (7, N = 8,400) = 2259.3, p < 0.001
Disgust	χ^2 (7, N = 8,400) = 1222.5, p < 0.001
Fear	χ^2 (7, N = 8,400) = 1266.9, p < 0.001
Joy	χ^2 (7, N = 8,400) = 164.86, p < 0.001
Sadness	χ^2 (7, N = 8,400) = 531.6, p < 0.001
Neutral	χ^2 (7, N = 8,400) = 113.96, p < 0.001
