# Supplementary material for: Unfolding and dynamics of affect bursts decoding in humans
Source: PLoS One. 2018 Oct 30;13(10):e0206216. doi: 10.1371/journal.pone.0206216 (PMC6207317; doi:10.1371/journal.pone.0206216)
Supplement: S5 Table — Mean and Standard Deviation of the Gate Durations for Each Emotion Separately. (PDF) [file pone.0206216.s012.pdf]

Mean and Standard Deviation of the Gate Durations for Each Emotion Separately

Gate	Anger	Disgust	Fear	Joy	Neutral	Sadness
10%	*M* = 88, *SD* = 47	*M* = 101, *SD* = 57	*M* = 73, *SD* = 31	*M* = 138, *SD* = 105	*M* = 82, *SD* = 36	*M* = 126, *SD* = 85
20%	*M* = 199, *SD* = 101	*M* = 241, *SD* = 141	*M* = 129, *SD* = 66	*M* = 344, *SD* = 249	*M* = 158, *SD* = 77	*M* = 306, *SD* = 194
30%	*M* = 340, *SD* = 153	*M* = 413, *SD* = 222	*M* = 226, *SD* = 104	*M* = 625, *SD* = 394	*M* = 288, *SD* = 123	*M* = 473, *SD* = 297
40ù	*M* = 487, *SD* = 223	*M* = 593, *SD* = 292	*M* = 329, *SD* = 129	*M* = 870, *SD* = 541	*M* = 421, *SD* = 164	*M* = 714, *SD* = 403
50%	*M* = 573, *SD* = 278	*M* = 660, *SD* = 392	*M* = 391, *SD* = 181	*M* = 1007, *SD* = 706	*M* = 501, *SD* = 190	*M* = 810, *SD* = 508
60%	*M* = 763, *SD* = 333	*M* = 852, *SD* = 468	*M* = 507, *SD* = 199	*M* = 1358, *SD* = 846	*M* = 673, *SD* = 253	*M* = 1140, *SD* = 621
70%	*M* = 889, *SD* = 409	*M* = 1089, *SD* = 521	*M* = 616, *SD* = 239	*M* = 1680, *SD* = 997	*M* = 810, *SD* = 313	*M* = 1319, *SD* = 724
80%	*M* = 1014, *SD* = 469	*M* = 1209, *SD* = 649	*M* = 660, *SD* = 285	*M* = 1785, *SD* = 1135	*M* = 890, *SD* = 350	*M* = 1488, *SD* = 882
90%	*M* = 1123, *SD* = 515	*M* = 1346, *SD* = 733	*M* = 751, *SD* = 333	*M* = 2103, *SD* = 1313	*M* = 1006, *SD* = 392	*M* = 1724, *SD* = 951
100%	*M* = 1244, *SD* = 603	*M* = 1356, *SD* = 781	*M* = 824, *SD* = 376	*M* = 2325, *SD* = 1552	*M* = 1146, *SD* = 427	*M* = 1701, *SD* = 1087
