# Supplementary material for: Unfolding and dynamics of affect bursts decoding in humans
Source: PLoS One. 2018 Oct 30;13(10):e0206216. doi: 10.1371/journal.pone.0206216 (PMC6207317; doi:10.1371/journal.pone.0206216)
Supplement: S6 Table — Comparisons between general linear mixed models using the PCs computed with the principal component analysis on each emotion separately. (PDF) [file pone.0206216.s013.pdf]

Comparisons Between General Linear Mixed Models Using the PCs Computed With the Principal Component Analysis on Each Emotion Separately

		Chi-squared test, effect size, model fit
All	PC1×EMO	$\chi^2(14, N = 8{,}022) = 107.94$, $p < 0.001$, $R^2m = 0.42$, $R^2c = 0.49$, AIC = -2480.5, BIC = -2382.7
	PC2×EMO	$\chi^2(14, N = 8{,}022) = 39.59$, $p < 0.001$, $R^2m = 0.43$, $R^2c = 0.50$, AIC = -2560.8, BIC = -2462.9
	PC3×EMO	$\chi^2(14, N = 8{,}022) = 146.8$, $p < 0.001$, $R^2m = 0.43$, $R^2c = 0.50$, AIC = -2501.1, BIC = -2403.2
	PC4×EMO	$\chi^2(14, N = 8{,}022) = 98.87$, $p < 0.001$, $R^2m = 0.42$, $R^2c = 0.49$, AIC = -2462.9, BIC = -2365.1
Anger	PC1	$\chi^2(4, N = 1{,}357) = 29.18$, $p < 0.001$, $R^2m = 0.016$, $R^2c = 0.28$, AIC = -278.5, BIC = -257.6
	PC1×DUR	$\chi^2(10, N = 1{,}357) = 17.18$, $p = 0.01$, $R^2m = 0.226$, $R^2c = 0.50$, AIC = -725.2, BIC = -673.1
	PC2	$\chi^2(4, N = 1{,}357) = 37.69$, $p < 0.001$, $R^2m = 0.020$, $R^2c = 0.29$, AIC = -287.0, BIC = -266.1
	PC2×DUR	$\chi^2(10, N = 1{,}357) = 11.50$, $p = 0.022$, $R^2m = 0.224$, $R^2c = 0.50$, AIC = -718.5, BIC = -666.3
	PC3	$\chi^2(4, N = 1{,}357) = 98.91$, $p < 0.001$, $R^2m = 0.053$, $R^2c = 032$, AIC = -348.2, BIC = -327.4
	PC3×DUR	$\chi^2(10, N = 1{,}357) = 9.49$, $p = 0.028$, $R^2m = 0.223$, $R^2c = 0.50$, AIC = -715.6, BIC = -663.5
	PC4	*ns*
	PC4×DUR	*ns*
Disgust	PC1	$\chi^2(4, N = 714) = 12.81$, $p < 0.001$, $R^2m = 0.013$, $R^2c = 0.30$, AIC = 207.48, BIC = 225.7
	PC1×DUR	ns
	PC2	*ns*
	PC2×DUR	*ns*
	PC3	$\chi^2(4, N = 714) = 13.56$, $p < 0.001$, $R^2m = 0.014$, $R^2c = 0.30$, AIC = 206.7, BIC = 225.0
	PC3×DUR	ns
	PC4	$\chi^2(4, N = 714) = 26.96$, $p < 0.001$, $R^2m = 0.028$, $R^2c = 0.31$, AIC = 193.3, BIC = 211.6
	PC4×DUR	$\chi^2(10, N = 714) = 8.84$, $p = 0.031$, $R^2m = 0.11$, $R^2c = 0.39$, AIC = 117.9, BIC = 163.7
Fear	PC1	$\chi^2(4, N = 892) = 43.03$, $p < 0.001$, $R^2m = 0.034$, $R^2c = 0.34$, AIC = -284.2, BIC = -265.0
	PC1×DUR	ns
	PC2	*ns*
	PC2×DUR	*ns*
	PC3	*ns*
	PC3×DUR	*ns*
	PC4	$\chi^2(4, N = 892) = 26.74$, $p < 0.001$, $R^2m = 0.021$, $R^2c = 0.33$, AIC = -267.9, BIC = -248.7
	PC4×DUR	*ns*
Joy	PC1	$\chi^2(4, N = 2026) = 52.16$, $p < 0.001$, $R^2m = 0.014$, $R^2c = 0.36$, AIC = -2128.0, BIC = -2105.6
	PC1×DUR	*ns*
	PC2	$\chi^2(4, N = 2026) = 151.21$, $p < 0.001$, $R^2m = 0.050$, $R^2c = 0.38$, AIC = -2237.1, BIC = -2214.6
	PC2×DUR	*ns*
	PC3	*ns*
	PC3×DUR	*ns*
	PC4	$\chi^2(4, N = 2026) = 51.82$, $p < 0.001$, $R^2m = 0.017$, $R^2c = 0.36$, AIC = -2137.7, BIC = -2115.2
	PC4×DUR	*ns*

Neutral	PC1	*ns*
	PC1×DUR	*ns*
	PC2	χ^2(4, *N* = 1,410) = 34.28, $p < 0.001$, R^2m = 0.017, R^2c = 0.30, AIC = -1268.8, BIC = -1247.7
	PC2×DUR	*ns*
	PC3	χ^2(4, *N* = 1,410) = 37.95, $p < 0.001$, R^2m = 0.019, R^2c = 0.30, AIC = -1272.4, BIC = -1251.4
	PC3×DUR	*ns*
	PC4	*ns*
	PC4×DUR	*ns*
Sadness	PC1	*ns*
	PC1×DUR	*ns*
	PC2	χ^2(4, *N* = 1,623) = 81.16, $p < 0.001$, R^2m = 0.023, R^2c = 0.56, AIC = -1175.2, BIC = -1153.7
	PC2×DUR	*ns*
	PC3	*ns*
	PC3×DUR	*ns*
	PC4	*ns*
	PC4×DUR	χ^2(10, *N* = 1,623) = 10.15, $p = 0.028$, R^2m = 0.06, R^2c = 0.61, AIC = -1312.0, BIC = -1258.1

Note. PC1×EMO means that both PC1 and EMOTION are used as fixed effect as well as the interaction between them. Chi-squared test, effect size, Akaike information criterion (AIC), and Bayesian information criterion (BIC) obtained when comparing individual model with PC scores for a specific emotion as a fixed effect and a model with no fixed effect (only the participants as a random effect). All the p-values are FDR-corrected for multiple comparisons.
